# Game Design, Effectiveness, and Implementation of Serious Games Promoting Aspects of Mental Health Literacy Among Children and Adolescents: Systematic Review

**DOI:** 10.2196/67418

**Published:** 2025-05-05

**Authors:** Michael Zeiler, Sandra Vögl, Ursula Prinz, Nino Werner, Gudrun Wagner, Andreas Karwautz, Natalie Zeller, Lorenz Ackermann, Karin Waldherr

**Affiliations:** 1 Medical University of Vienna, Department for Child and Adolescent Psychiatry Vienna Austria; 2 Ferdinand Porsche FERNFH - Distance Learning University of Applied Sciences Wiener Neustadt Austria

**Keywords:** serious games, gamification, mental health literacy, game design, children, adolescents, systematic review, artificial intelligence

## Abstract

**Background:**

The effects of traditional health-promoting and preventive interventions in mental health and mental health literacy are often attenuated by low adherence and user engagement. Gamified approaches such as serious games (SGs) may be useful to reach and engage youth for mental health prevention and promotion.

**Objective:**

This study aims to systematically review the literature on SGs designed to promote aspects of mental health literacy among adolescents aged 10 to 14 years, focusing on game design characteristics and the evaluation of user engagement, as well as efficacy, effectiveness, and implementation-related factors.

**Methods:**

We searched PubMed, Scopus, and PsycINFO for original studies, intervention development studies, and study protocols that described the development, characteristics, and evaluation of SG interventions promoting aspects of mental health literacy among adolescents aged 10 to 14 years. We included SGs developed for both universal and selected prevention. Using the co.LAB framework, which considers aspects of learning design, game mechanics, and game design, we coded the design elements of the SGs described in the studies. We coded the characteristics of the evaluation studies; indicators of efficacy, effectiveness, and user engagement; and factors potentially fostering or hindering the reach, efficacy and effectiveness, organizational adoption, implementation, and maintenance of the SGs.

**Results:**

We retrieved 1454 records through database searches and other sources. Of these, 36 (2.48%) studies describing 17 distinct SGs were included in the review. Most of the SGs (14/17, 82%) were targeted to a universal population of youth, with learning objectives mainly focusing on how to obtain and maintain good mental health and on enhancing help-seeking efficacy. All SGs were single-player games, and many (7/17, 41%) were embedded within a wider pedagogical scenario. Diverse game mechanics and game elements (eg, minigames and quizzes) were used to foster user engagement. Most of the SGs (12/17, 71%) featured an overarching storyline resembling real-world scenarios, fictional scenarios, or a combination of both. The evaluation studies provided evidence for the short-term efficacy and effectiveness of SGs in improving aspects of mental health literacy as well as their feasibility. However, the evidence was mostly based on small samples, and user adherence was sometimes low.

**Conclusions:**

The results of this review may inform the future development and implementation of SGs for adolescents. Intervention co-design, the involvement of facilitators (eg, teachers), and the use of diverse game mechanics and customization to meet the needs of diverse users are examples of elements that may promote intervention success. Although there is promising evidence for the efficacy and effectiveness of SGs for promoting mental health literacy in youth, there is a need for more rigorously planned studies, including randomized controlled trials and real-world evaluations, that involve follow-up measures and the assessment of in-game performance alongside self-reports.

## Introduction

### Background

It is well established that mental health problems are common among adolescents [1-3], a situation further exacerbated by the COVID-19 pandemic [4-6]. Almost 50% of psychological disorders develop before the age of 14 years [7]. Current research suggests that—apart from full-syndrome psychiatric disorders—subclinical mental health problems in adolescents are on the rise, which underscores the public health relevance of this topic [8,9]. Consequently, implementing effective strategies to tackle psychological distress at an early stage is now regarded as one of the top priorities [10]. According to the World Health Organization, adolescence, defined as the age range between 10 and 19 years, is the perfect time for the prevention of mental health problems not only because most problems have their onset during this stage of development but also due to the high neuroplasticity characteristic of the adolescent brain [11]. Furthermore, mental health is much more than the absence of mental illness. Therefore, it is suggested that it is not only important to prevent the onset of mental health disorders but also to actively promote positive mental health [12]. The Sustainable Development Goals of the United Nations include the goal of reducing premature mortality from noncommunicable diseases by one-third by 2030 [11], underlining the importance of mental health promotion and mental illness prevention programs.

One approach to achieving this goal is to promote mental health literacy [13]. This term originated in health literacy but has since developed into a domain-specific construct and evolved over time [14,15]. Most recent definitions include the following components: “understanding how to obtain and maintain positive mental health; understanding mental disorders and their treatments; decreasing stigma related to mental disorders; and enhancing help-seeking efficacy (knowing when and where to seek help and developing competencies designed to improve one’s mental health care and self-management capabilities)” [15].

A recent survey on health behavior in school-age children in Austria [16] showed that a substantial proportion of those aged 11 to 17 years (31% of the girls and 24% of the boys) reported low levels of self-efficacy. Furthermore, mental health literacy seemed to be particularly low, with only approximately half of those aged 11 to 17 years reporting that they knew where to find support for themselves or relatives in the event of mental health problems [16]. These findings underline the need to promote mental health literacy in adolescents, particularly focusing on competencies related to maintaining positive mental health, self-management strategies in case of mild psychological problems, and when to involve parents or other caregivers for more severe issues. Given the many physical and mental changes that occur during early adolescence, this period offers a window of opportunity to promote mental health literacy. It has been shown that better mental health literacy during adolescence can positively affect mental health outcomes in adulthood [17]. Consequently, developing good mental health literacy during adolescence seems imperative for becoming a healthy adult.

Digital (eHealth) interventions to promote mental health literacy have been regarded as useful and attractive [15]. However, the advantages and effectiveness of traditional psychoeducational eHealth interventions are often attenuated by low adherence and high dropout rates, particularly in the field of prevention and health promotion [18-20]. Recent research has shown that various stakeholders advocate for more game-based and implicit learning approaches [21]. For adolescents especially, serious games (SGs) are considered promising [18,20,22,23]. An SG is typically a computer- or smartphone-delivered intervention that uses gaming as the primary medium to educate and change behavior [24]. An SG aimed at promoting mental health literacy might simulate real-life scenarios where players make decisions that impact a character’s mental well-being, such as managing stress or identifying mental health problems in game avatars. In many cases, the SG is embedded in an overarching storyline (eg, an adventure mission) and might include dialogues with nonplayer characters, minigames, and in-game feedback to educate players while reinforcing positive coping strategies. SGs are considered beneficial for the following reasons: (1) they may appeal to adolescents who might otherwise not use a mental health intervention, thereby positively impacting reach; (2) the combination of fun and motivational elements can improve engagement; and (3) various mechanisms can be used to achieve the goals of the intervention [24]. In this context, the use of SGs represents a promising and innovative strategy to promote mental health literacy.

As a recently published review has shown, the majority of existing SGs targeting mental health literacy were designed for people with existing mental health problems [18], while only a few were designed to promote mental health literacy in a universal population. Furthermore, most of the identified SGs were designed for adults rather than adolescents. Nonetheless, many of these SGs have been shown to be potentially effective in improving mental health, and high levels of user satisfaction were reported [18,25], although heterogeneity in effects was also observed. Differences in design elements, including learning objectives, game structures, game mechanics, in-game interactions, and implementation strategies, may influence user engagement and effectiveness.

The co.LAB framework [26] provides a structured overview of the fundamental design elements to be considered when developing an SG. It includes aspects of learning design, learning and game mechanics, and game design (refer to the Methods section for more details) and also addresses the interplay between these elements. An in-depth consideration of these aspects is crucial for user engagement and game effectiveness. To date, no systematic review has examined SGs that target mental health literacy in adolescents and specifically focus on game design elements.

### Objectives

The objective of this study was to conduct a systematic review of SGs aimed at promoting aspects of mental health literacy in adolescents aged 10 to 14 years. In contrast to the systematic review by Ferrari et al [18], this study focuses on a detailed review of game design elements based on the co.LAB framework [26] and includes aspects related to implementation in real-world settings. Moreover, this study focuses exclusively on SGs designed for nonclinical samples. The rationale for focusing on the narrow age range (10-14 y) is 2-fold: first, as outlined previously, early adolescence is a critical period for the development of mental health problems; thus, preventive interventions may be particularly important. Second, the results from this systematic review will directly inform the development of a prototype of a new SG aimed at promoting mental health literacy in adolescents [27]. The age range of 10 to 19 years was considered too broad to develop an SG that would appeal equally to, and be appropriate for, those aged 10 to 11 years and those aged 18 to 19 years.

Specifically, the following research questions (RQs) were developed:

1. Which design elements (learning design, mechanics, and game design) have been used in existing SGs to promote mental health literacy?
2. What is known about the efficacy and effectiveness of these games, as well as user engagement with them?
3. What factors have fostered or hindered reaching the target population and adoption by organizations, as well as the efficacy and effectiveness, implementation, and maintenance of these interventions?

## Methods

### Overview

We conducted a systematic review following the PRISMA (Preferred Reporting Items for Systematic Reviews and Meta-Analyses) guidelines [28]. The PRISMA checklist is presented in Multimedia Appendix 1. This review was not registered, and no protocol was published in advance.

### Eligibility Criteria

The eligibility criteria for the inclusion of studies in the review are presented in Textbox 1.

**Eligibility criteria.**

**Inclusion criteria**

1. The study had to be related to either the development or evaluation of a mental health literacy intervention. We included studies targeting mental health literacy in general or at least 1 aspect of it as defined by Kutcher et al [15]. These aspects may include competencies to maintain or obtain positive mental health, recognize the symptoms of mental disorders, and deal with mental health issues (eg, stress coping, emotion regulation, and dealing with negative emotions), as well as knowledge of services for seeking help and the destigmatization of mental problems or disorders.
2. The intervention had to use a game-based approach, involving games playable on a PC, smartphone, or tablet. Both game-only and blended interventions (ie, those combining a serious game with face-to-face sessions) were included. However, the game had to be the primary component of the intervention. Digital interventions that merely incorporated single gamification elements into nongame contexts were excluded.
3. Studies were included if the intervention was designed for either universal prevention or selected prevention (ie, groups considered to be at risk). In this regard, we referred to the stepped care model for video game interventions formulated by Ferrari et al [18], who defined “step 0” as population-based interventions for mental health prevention and promotion as well as education for asymptomatic youth (ie, universal prevention) and “step 1” as interventions targeted to groups considered to be at risk or those with suspected mental health problems. However, studies using mixed samples (eg, healthy individuals and those with a mental illness) were included if the intervention was primarily targeted to healthy individuals.
4. The described target population of the game had to comprise adolescents aged 10 to 14 years.
5. The study had to be an evaluation study, usability and feasibility study, or study protocol that described the development or design of an intervention or the design of a randomized controlled trial.
6. The study had to be published in a peer-reviewed journal.
7. It had to be written in English or German (languages understood by the research team).
8. The study had to be published in 2013 or later (within the past 10 years). By focusing on studies from the last 10 years, we aimed to avoid the inclusion of serious games based on outdated technology.

**Exclusion criteria**

1. Reviews, conference abstracts, books and book chapters, and theses were excluded.
2. Studies on games that involved virtual reality, augmented reality, or biofeedback were also excluded because these types of interventions require specific equipment and are usually not designed to be widely disseminated, such as for use in schools.

### Information Sources and Search Strategy

We searched 3 electronic databases (PubMed, Scopus, and PsycINFO) for relevant literature. The selected keywords were related to mental health (“mental health OR mental health literacy OR mental health promotion OR coping OR stress management OR emotion recognition OR emotion regulation OR stigma”) and gamified approaches (“serious game* OR digital game* OR serious video game* OR game-based OR gamification OR smartphone game* OR mobile game*”). These 2 groups of keywords were combined using an “AND” operator. The exact search syntax is provided in Table S1 in Multimedia Appendix 2 [22,23,29-53]. In addition, we searched the reference lists of relevant reviews and included studies to identify additional publications.

### Selection Process

All studies retrieved through the literature search were imported into a reference manager. In the first step, we excluded duplicates, reviews, books and book chapters, theses, conference abstracts, and studies published before 2013. Next, 2 pairs of independent researchers (UP and SV; MZ and SV) screened titles and abstracts for inclusion criteria. To establish a shared understanding of eligibility, the first 15 titles and abstracts were evaluated by all reviewers and discussed immediately afterward. After abstract screening, interrater reliability was 93.5%. Articles that passed the abstract screening and those retrieved through other sources (eg, reference lists and reviews) underwent full-text review by the same pairs of independent researchers. Any disagreements were discussed with a third researcher (KW) until consensus was reached. Interrater reliability for the full-text screening was 91.8%.

### Data Collection Process and Coding Procedure

We collaboratively developed the coding sheet, which included the extraction of design elements of the SGs as well as study characteristics and results. The coding of intervention design elements was based on the co.LAB framework [26], which categorizes design elements across three dimensions: (1) learning design (including learning profiles, learning functions, learning objectives, learning foundations, knowledge foundations, and pedagogical scenarios), (2) mechanics (including learning mechanics, game mechanics, learning and game incentives and rewards, and learning and game interactions), and (3) game design (including goals and rules, game universe, fidelity and simulation models, interfaces and user experience, and game structure and narrative). Definitions of these items can be found in Table S2 in Multimedia Appendix 2.

Furthermore, regarding study characteristics, we extracted the publication year, language, country in which the intervention was conducted, and study arms. Regarding sample characteristics, we coded the inclusion and exclusion criteria, sample size, age, sex, recruitment and implementation setting, follow-up time points, outcome variables, analysis methods, main results, and adherence and dropout measures. In addition, we extracted potentially hindering or fostering factors related to participant reach and intervention effectiveness as well as organizational adoption, implementation, and maintenance of the intervention, as outlined in the RE-AIM (Reach, Effectiveness, Adoption, Implementation, and Maintenance) framework [54], if these were discussed in the included papers. We included not only fostering or hindering factors identified through systematic evaluation studies on SGs but also factors that the authors described as potentially relevant in the discussion sections of the papers. A full overview of the coded items is provided in Table S2 in Multimedia Appendix 2.

To establish a common understanding of how to use the coding sheet appropriately, 2 full texts were coded independently by 4 researchers (UP, SV, MZ, and KW) and subsequently discussed. The extraction and coding of all remaining studies were performed independently by 2 researchers (UP and SV). Any inconsistencies were discussed with at least 1 additional researcher (MZ or KW) until consensus was reached.

Furthermore, we used the National Institutes of Health quality assessment tool [55] for controlled intervention studies (14 items) or for pretest-posttest studies with no control group (12 items) to evaluate the quality of the included evaluation studies. The critical appraisal of the included studies was also performed independently by 2 of the following researchers (MZ, SV, UP, and LA), and any inconsistent results were discussed with a third researcher (KW).

### Data Synthesis

We conducted a narrative synthesis of the coded study and game characteristics as well as the results regarding efficacy and effectiveness. The results were summarized in tables, which are presented in the Results section or in the multimedia appendices. Where applicable, we counted the frequencies of relevant categories (eg, the number of stand-alone vs blended interventions, the number of SGs based on fictional vs real-world scenarios, and the number of interventions implemented in schools vs other settings) and reported them in the text. Due to the substantially diverse outcome measures used in the individual studies, we refrained from conducting a meta-analysis to evaluate the efficacy and effectiveness of SGs. Fostering and hindering factors based on the RE-AIM framework were extracted and categorized and are briefly summarized in the text.

## Results

### Literature Search Process

**Figure 1:**
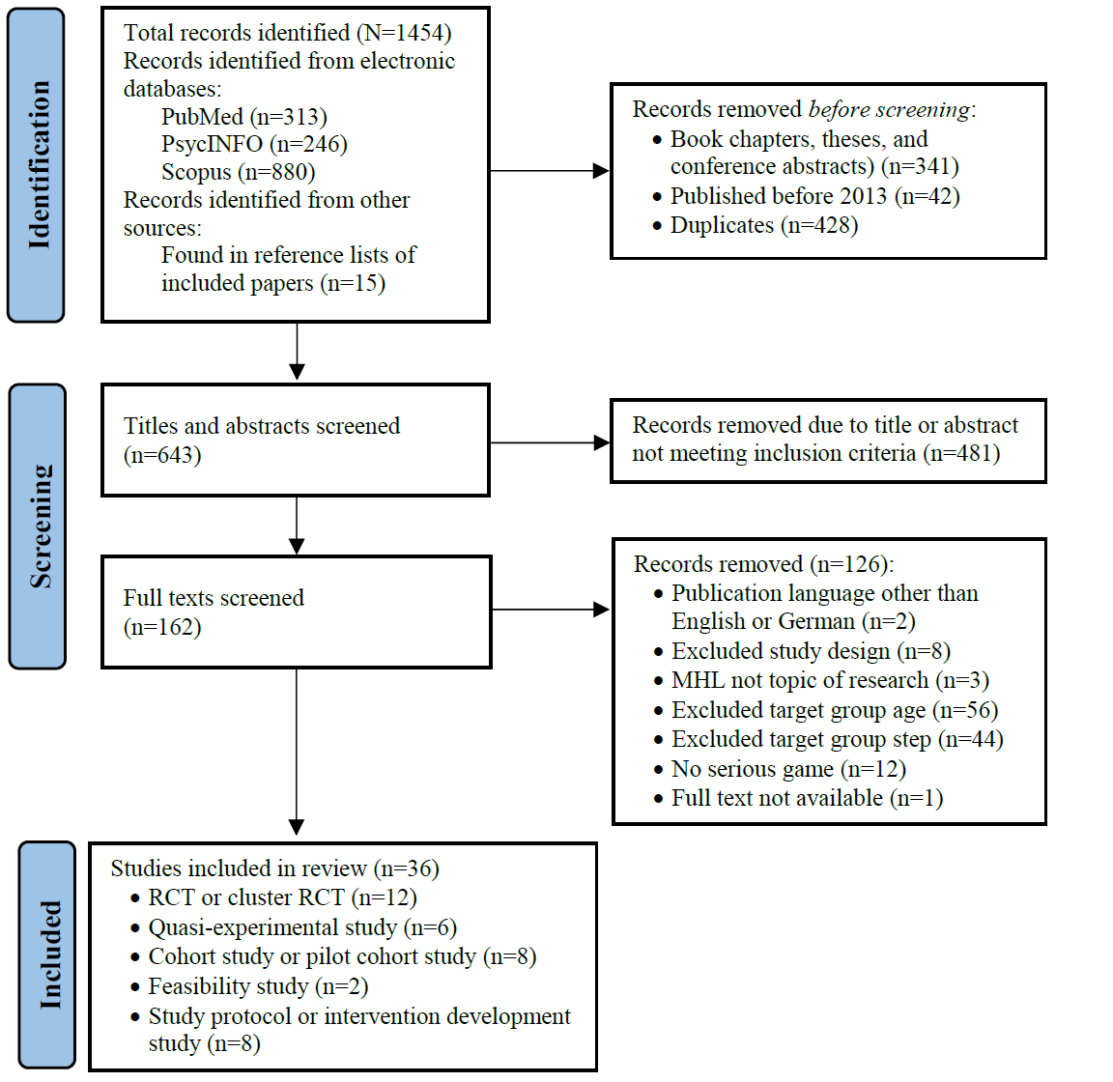
PRISMA (Preferred Reporting Items for Systematic Reviews and Meta-Analyses) flowchart documenting the literature search process. MHL: mental health literacy; RCT: randomized controlled trial.

The literature search, completed on July 13, 2023, yielded 1439 results (PubMed: n=313, 21.75%; PsycINFO: n=246, 17.1%; and Scopus: n=880, 61.15%). An additional 15 studies were identified through other sources. After removing duplicates, book chapters, theses, conference abstracts, and studies published before 2013, a total of 643 (44.22%) of the 1454 studies remained. The titles and abstracts of these papers were screened for inclusion criteria. Studies that passed the screening (162/643, 25.2%) underwent full-text review, of which 22 (13.6%) were excluded for not meeting the inclusion criteria regarding publication language, study type, and SG intervention. Of the remaining 140 studies, 103 (73.6%) were excluded because they did not focus on mental health literacy or described SGs not developed for our defined target group, while 1 (0.7%) study was excluded because the full text was not available. Finally, of the initially identified 1454 studies, 36 (2.48%) were included in this review (refer to Figure 1 for the PRISMA flowchart).

### Characteristics of Included Studies

An overview of the study and sample characteristics of the included studies as well as brief descriptions of the SGs are provided in Table 1. Among the 36 studies, we identified 12 (33%) randomized controlled trials (RCTs) or cluster RCTs, 6 (17%) quasi-experimental studies, 8 (22%) cohort studies (including pilot studies) without a control group, and 2 (6%) feasibility studies, while 8 (22%) were study protocols or papers describing the intervention development process. Altogether, these papers described the design and evaluation of 17 SGs aimed at promoting aspects of mental health literacy. Of note, 7 (19%) of the 36 articles focused on the *REThink* intervention. However, of these 7 articles, 4 (57%) [29-32] referred to the same sample or a subset of the sample included in the overarching RCT [33]. In addition, 2 (6%) [34,35] of the 36 articles focused on the *Happy* intervention, but both merely repeated the findings of 2 other articles [36,37].

**Table 1:** Study and sample characteristics.

Serious game and study; country		Study design (study arms)	Inclusion and exclusion criteria	Sample size (size of each group); participation rate (%)	Age (y), mean (SD; range)	Girls (%)	Implementation setting
**Adventures Aboard the S.S. Grin (level of prevention: step 1^a^): the child’s avatar joins the crew of a sailing ship and travels around an island, interacting with nonplayer characters and solving social challenges to strengthen social and emotional skills (eg, emotion regulation, perspective taking, and impulse control)**							
	Sanchez et al [39], 2017; United States	RCT^b^ (IG^c^ vs waitlist CG^d^)	Inclusion criteria: aged 7-11 y, internet access, proficiency in English, and social skills deficits	69 (33 and 36); NR^e^	NR (NR; 7-11)	NR	Home
**Aislados** **(level of prevention: step 0^f^): characters are deployed in an imaginary boat traveling to some newly discovered islands; the player has to interact with other nonplayer characters and obtain information for task achievement or solving riddles related to socioemotional skills (eg, assertiveness, emotional skills, and decision-making)**							
	Cejudo et al [40], 2020; Spain	Quasi-experimental (IG vs waitlist CG)	Inclusion criterion: parental consent; exclusion criteria: disciplinary problems, special educational needs, intellectual disability, and <75% of intervention completed	187 (97 and 90); NR	13.8 (1.6; 12-17)	53	School
**Emodiscovery** **(level of prevention: step 0): different emotionally challenging scenarios for nonplayer characters are presented in the game; the player has to recognize the emotions of these characters and interact with them to make them feel better**							
	Pacella and López-Pérez [42], 2018; United Kingdom	Cohort study (IG only)	Inclusion criterion: parental consent	58 (N/A^g^); 90	8.8 (1.0; 8-11)	36.2	School
	López-Pérez and Pacella [38], 2021; United Kingdom and Spain	Cohort study (IG only)	Inclusion criterion: parental consent	208 (United Kingdom: 100; Spain: 108); >95	United Kingdom: 9.1 (0.83; 8-10); Spain: 9.0 (0.83; 8-10)	United Kingdom: 39; Spain: 44	School
**EmoTIC (level of prevention: step 0): the player has crashed on a fictional planet, and the main objective is to return to earth, which can be achieved by solving different tasks related to the use of socioemotional skills, including stress-coping strategies, assertiveness, and self-efficacy**							
	de la Barrera et al [56], 2021; Spain	Study protocol	Inclusion criteria: first or second year of compulsory secondary education, mobile device with internet access, and parental consent; exclusion criteria: aged <11 y and >16 y	N/A	N/A	N/A	N/A
	de la Barrera et al [49], 2021; Spain	RCT (IG vs waitlist CG)	Inclusion criteria: first or second year of compulsory secondary education, mobile device with internet access, and parental consent; exclusion criteria: aged <11 y and >16 y and not completed ≥80% of the pretest assessment	286 (119 and 167); NR	IG: 12.6 (0.7; 11-15); CG: NR	IG: 42.9; CG: NR	School and home
**Happy 8-12 and Happy 12-16 (level of prevention: step 0): different examples of everyday conflicts are presented; the player has to identify the emotions of nonplayer characters and respond to conflict situations by suggesting adaptive emotion regulation strategies**							
	Filella et al [36], 2016; Spain (information repeated in Ros-Morente et al [35], 2018 and Filella and Ros-Morente [34], 2023)	Quasi-experimental cluster trial (IG vs waitlist CG)	NR	574 (351 and 223); NR	10.5 (0.7; NR)	47.6	School
	Filella et al [37], 2018; Spain (information repeated in Ros-Morente et al [35], 2018 and Filella and Ros-Morente [34], 2023)	Quasi-experimental cluster trial (IG vs waitlist CG)	Inclusion criterion: parental oral consent	903 (472 and 431); NR	12.6 (0.6; NR)	47.8	School
**IMPeTUs (level of prevention: step 0): the player has to navigate through different mental health–related challenges and make decisions reflecting the level of mental health literacy skills as well as anxiety- and depression-focused self-management skills; the game also includes minigames focusing on improving specific coping skills (eg, distraction and breathing techniques, journaling, and talking about mental health issues)**							
	Brooks et al [57], 2021; Indonesia	Study describing intervention development	N/A	N/A	N/A	N/A	N/A
	Brooks et al [50], 2023; Indonesia	Pilot usability and feasibility study (IG only)	Inclusion criteria: aged 11-15 y, in contact with one of the defined study locations, and adolescents’ assent and parental consent	78 (N/A); NR	12.9 (1.4; 11-15)	48.7	School, community, and health care organizations
**Moving Stories (level of prevention: step 0): an avatar with the symptoms of depression is introduced, and player is asked to help her to feel better through different actions, achieved by applying skills related to the correct identification of depressive symptoms, knowledge of help-seeking options, and first aid skills**							
	Gijzen et al [58], 2021; Netherlands	Implementation and feasibility qualitative study (IG only)	Inclusion criteria: enrolled in the second year of high school and sufficiently fluent in Dutch (the schools concerned were participating in the overall STORM^h^ program)	982 (NR); 52	NR (NR; 11-15)	NR	School and home
	Tuijnman et al [59], 2019; Netherlands	Study protocol paper	Exclusion criterion: refusal to participate by parents or adolescent	N/A	N/A	N/A	N/A
	Tuijnman et al [43], 2022; Netherlands	Cluster RCT (IG vs waitlist CG)	Exclusion criterion: refusal to participate by parents or adolescent	185 (99 and 86); 67.3	13.4 (0.7; 12-15)	Total: 43.3; IG: 34; CG: 58	School
**Unnamed serious game (level of prevention: step 0): the player is transported into a fantasy world and has to solve the mystery of a catastrophic event (almost all people have vanished from a virtual school); the mystery is solved by understanding interpersonal conflicts and applying adaptive interpersonal emotion regulation skills while interacting with nonplayer characters**							
	Mittmann et al [60], 2021; Austria and United Kingdom	Study protocol for RCT (IG vs active CG)	Inclusion criteria: aged 10-14 y, parental consent, willingness to complete pretest and posttest questionnaire and play the game in between, able to read and understand German or English, and access to an internet-enabled device	180 (planned; NR); NR	NR	NR	School and home
**POD Adventures (level of prevention: step 1): short vignette-based stories related to common stressors of nonplayer characters are presented, and the player has to react accordingly to cope with these stressors; moreover, the player is guided through problem-solving steps for their own problems**							
	Gonsalves et al [61], 2019; India	Intervention development study and user testing (IG only)	Inclusion criteria: had to speak English, Hindi, or Konkani; and parental consent	50 (N/A); NR	14.5 (NR; 12-17)	56	School
	Gonsalves et al [46], 2021; India	Pilot cohort study (IG only with 2 delivery formats)	Inclusion criteria: students in grades 9-12, self-referral for psychological help with perceived stress, and proficient in English and Konkani; exclusion criterion: elevated risk of self-harm or suicide	248 (NR); 14.1	15.6 (NR; 13-19)	50	School
	Gonsalves et al [62], 2021; India	Study protocol	Inclusion criteria: students in grades 9-12, access to an internet-enabled Android smartphone, English proficiency, and students’ assent and parental consent; exclusion criteria: unable to understand intervention material and elevated risk for self-harm or suicide and requiring external referral	N/A	N/A	N/A	N/A
	Gonsalves et al [47], 2023; India	Pilot RCT (IG vs CG, including enhanced usual care)	Inclusion criteria: students in grades 9-12, access to an internet-enabled Android smartphone, English proficiency, and students’ assent and parental consent; exclusion criteria: unable to understand intervention material and elevated risk for self-harm or suicide and requiring external referral	11 (5 and 6); <1	Total: 15.3 (1.0; NR); IG: 15.0 (0.5; 13-19); CG: 15.5 (1.3; 13-19)	Total: 63.6; IG: 60; CG: 66.7	Home (school recruitment)
**Professor Gooley and the Flame of Mind (level of prevention: step 0): the player takes the role of a space intern in a fictional setting where cognitive distortions prevail on earth; during a space journey to 8 fictional planets, the player completes quests that require skills related to, for example, the identification of automatic thoughts, self-esteem, interpersonal communication, and problem-solving**							
	Huen et al [48], 2016; China	Cohort study (IG only)	NR	498 (N/A); 31	12.6 (1.2; NR)	50.6	NR (school recruitment)
**R.E.M.I. (level of prevention: step 0): each player is paired with a virtual robot and tasked with training it in various social and emotional skills**							
	Saleme et al [63], 2021; Australia	Intervention development study and study protocol	NR	NR	NR (NR; 8-11)	NR	School and home
**RegnaTales (level of prevention: step 0): a series of 6 different game-based mobile apps through which players learn to improve anger management by identifying feelings and bodily reactions, distinguishing positive from negative thoughts, applying anger-coping skills, and learning specific techniques (eg, cognitive restructuring, breathing, and perspective taking)**							
	Ong et al [44], 2019; Singapore	Pilot cohort study (IG only)	Inclusion criteria: typically developing children or children with disruptive behavior disorders, and children’s assent and parental consent	72 (N/A); 51.4 in the disruptive behavior disorders group	8.8 (1.7; 6-12)	31.9	Clinical setting
**REThink (level of prevention: step 0): the player’s mission is to help people on the planet escape the negative influence of an “Irrationalizer” by supporting them in becoming more rational and happier, which is achieved by performing tasks related to various mental health and coping skills (eg, replacing irrational cognitions, recognizing emotions, identifying problem-solving steps, and applying breathing techniques)**							
	David et al [51], 2018; Romania	Pilot cohort study (IG only)	Inclusion criterion: children’s assent and parental consent	25 (N/A); 88	13.4 (0.7; 10-16)	36	School
	David et al [33], 2019; Romania	RCT (IG vs face-to-face IG vs waitlist CG)	Inclusion criterion: informed consent from parents and school principal	165 (54, 55, and 56); 86	Total: 13.0 (2.1; 10-16); IG: 13.0 (2.1; NR); face-to-face IG: 12.8 (2.0; 10-16); CG: 12.9 (2.2; 10-16)	64	School
	David et al [29], 2019; Romania	RCT (IG vs face-to-face IG vs waitlist CG)	Inclusion criterion: informed consent from parents and school principal	165 (54, 55, and 56); 86	Total: 13.0 (2.1; 10-16); IG: 13.0 (2.1; NR); face-to-face IG: 12.8 (2.0; 10-16); CG: 12.9 (2.2; 10-16)	64	School
	David et al [23], 2021; Romania	Pilot cohort study (IG only)	Inclusion criterion: informed consent from parents and school authorities	31 (N/A); 100	12.3 (1.4; 10-16)	58	School
	David et al [31], 2022; Romania	RCT (IG vs face-to-face IG vs waitlist CG)	NR	165 (54, 55, and 56); 86^i^	12.8 (2.0; NR)	58.6	School
	David et al [30], 2022; Romania	Pilot cohort study (IG only)	Inclusion criterion: completion of initial assessment	48 (N/A); NR^j^	13 (2.1; 10-16)	75	School
	David and Magurean [32], 2022; Romania	RCT (IG only)	NR	34 (N/A); NR^k^	13 (2.1; 10-16)	75	School
**The Singularities (level of prevention: step 1): the player takes on the role of a “Singular,” a superhuman individual with special gifts, who resides in a school; the player is told that because of their uniqueness, Singulars face prejudice, often driven by fear and misunderstanding; the player is tasked with finding a team to help them complete their final mission, which is achieved by encouraging help-seeking behavior and the use of productive coping skills**							
	Coulter et al [64], 2019; United States	Study protocol	Inclusion criteria: English speaking, living in the United States, aged 14-18 y, experienced bullying or cyberbullying in the past year, sexual or gender minority identity, have access to a computer, and possess an email address	N/A	N/A	N/A	N/A
	Egan et al [45], 2021; United States	RCT (IG vs active CG)	Inclusion criteria: English speaking, living in the United States, aged 14-18 y, experienced (cyber)bullying in the past year, sexual or gender minority identity, have access to a computer, and possess an email address	240 (120 and 120); 59	Total: 15.8 (1.1; 14-18); IG: 15.8 (1.1; 14-18); CG: 15.7 (1.1; 14-18)	Cisgender girls, total: 16.3; IG: 15; CG: 18	Home
**Stigma-Stop (level of prevention: step 0): the player’s character visits nonplayer characters with mental disorders (eg, schizophrenia, depression, bipolar disorder, and panic disorder) to motivate them to participate in a video game design contest; this is achieved by understanding the symptoms of mental disorders; the game is complemented by minigames designed to enhance the understanding of mental disorders and reduce stigma**							
	Cangas et al [22], 2017; Spain	RCT (IG vs active CG)	Inclusion criterion: oral informed consent	552 (484 and 68); NR	15.8 (2.7; 14-18)	50	Laboratory (school recruitment)
	Cangas et al [41], 2019; Spain	RCT (IG vs active CG)	Inclusion criterion: oral informed consent	530 (412 and 118); NR	18.5 (4.3; 14-59)	61.5	Laboratory (school and university recruitment)
**The Adventures of DoReMiFa (level of prevention: step 0): the player lives on a fictional planet** **whose** **inhabitants are** **unhappy; in response, the president sends 4 monsters (Do, Re, MI, and Fa) to Earth to look for a book hidden in a primary school, which would help improve the mental health of their fellow citizens; while searching for this book, different challenges and tasks are performed requiring emotional competence as well as problem-solving and social skills**							
	Shum et al [52], 2019; China	Quasi-experimental trial (IG vs waitlist CG)	Inclusion criterion: informed consent from parents	459 (264 and 195); NR	IG: 9.5 (0.72; 8-12); CG: 9.5 (0.64; 8-12)	IG: 69; CG: NR	School
**Zoo U (level of prevention: step 0): the player has to interact with virtual teachers and classmates to learn about and take care of animals; this task requires various skills, including impulse control, communication, cooperation, social initiation, empathy, and emotion regulation**							
	Craig et al [53], 2016; United States	RCT (IG vs waitlist CG)	Inclusion criteria: aged 7-11 y, proficiency in English, and access to internet-enabled computer	47 (23 and 24); NR	9.7 (1.3; 7-11)	41	Home

^a^Selected prevention.

^b^RCT: randomized controlled trial.

^c^IG: intervention group.

^d^CG: control group.

^e^NR: not reported.

^f^Universal prevention.

^g^N/A: not applicable.

^h^STORM: Strong Teens and Resilient Minds.

^i^n=142 analyzed (subsample of David et al [29]).

^j^Subsample of intervention group of David et al [29].

^k^Subsample of intervention group of David et al [29]).

About a half of the SGs (8/17, 47%) were developed and evaluated in European countries, including Spain, the Netherlands, Romania, Austria, and the United Kingdom, SGs on this topic were also developed in Asian countries (Indonesia, India, China, and Singapore) as well as in Australia and the United States. Of note, only 2 (6%) of the 36 studies evaluated an SG or planned to evaluate it in >1 country [38,60].

Most of the studies (25/36, 69%) intended to include a broad population of participants by defining minimal inclusion and exclusion criteria, typically limited to parental consent, age range, access to a digital device for gameplay, and language proficiency. The target groups for most of the identified SGs (14/17, 82%) were a universal population of children or adolescents (step 0, universal prevention). Only 3 (18%) of the 17 SGs were designed as selected prevention interventions (step 1): *Adventures Aboard the S.S. Grin* [39], targeted to individuals with social skills difficulties; *POD Adventures* [61], targeted to adolescents at risk of anxiety, depression, and conduct problems; and *The* *Singularities* [64], developed for sexual and gender minority youth with previous experience of (cyber)bullying exposure. Most of the SGs (12/17, 71%) were designed for young adolescents within a narrow age range, specifically targeting individuals aged 10 to 14 years. However, some of the SGs (4/17, 24%) were additionally applied in adolescents aged >14 years [40,59,61,64]. *Stigma-Stop* [41] was additionally used in a university student population.

Sample sizes varied widely across the studies, ranging from 11 [62] to 982 [58] participants. The majority of the studies (11/36, 31%) included <100 participants, followed by those that included 100 to 300 participants (10/36, 28%), and studies with >300 participants (9/36, 25%). The remaining studies (mostly study protocol papers) did not report sample size (6/36, 17%). In most of the samples, the gender distribution was approximately balanced, with the proportion of girls ranging from one-third to two-thirds in the majority of the studies. Of note, no SG was evaluated exclusively in samples composed only of boys or only of girls. Some of the studies (14/36, 39%) reported the participation rate, defined as the percentage of individuals who provided informed consent out of all those approached. High participation rates were found for the *Emodiscovery* [38,42] and *REThink* [23,33] interventions; moderate rates for *Moving Stories* [43,58]*, RegnaTales* [44], and *The Singularities* [45]; and low rates for *POD Adventures* [46,47] and *Professor Gooley and the Flame of Mind* [48]*.*

Half of the SGs (18/36, 50%) were implemented in a school setting, while in some of the studies (7/36, 19%), gameplay took place at home or in a university laboratory. A few of the studies (5/36, 14%) used a combination of school-based and at-home implementation. The remaining studies (6/36, 17%) did not report on the implementation setting.

### Game Characteristics Based on the co.LAB Framework

A detailed overview of the characteristics of the identified SGs is provided in Multimedia Appendix 3 [22,23,29-49,51-53,56-64].

#### Learning Design

The learning objectives of the SGs are summarized in Table 2, based on the mental health literacy dimensions proposed by Kutcher et al [15]. The majority of the SGs (16/17, 94%) aimed at helping users understand how to obtain and maintain good mental health as well as enhance help-seeking efficacy. Specifically, the most frequently mentioned learning objectives included developing and improving interpersonal communication skills, expressing one’s own feelings, recognizing the emotions of others, applying emotion regulation strategies, and using stress-coping techniques. Of the 17 SGs, only 4 (24%) focused on or incorporated elements to improve users’ understanding of mental disorders (depression, anxiety disorders, schizophrenia, bipolar disorder, and addictions) and their treatments, and only 3 (18%) explicitly aimed to decrease stigma related to mental disorders.

The learning objectives of all SGs (17/17, 100%) were geared toward testing or applying players’ existing knowledge or skills and supporting knowledge and skills acquisition. Only 5 (29%) of the 17 games (*The* *Singularities* [64], *POD Adventures* [61], *RegnaTales* [44], *Adventures Aboard the S.S. Grin* [39], and *The Adventures of DoReMiFa* [52]) defined learning goals that explicitly aimed to prepare players to apply the learned skills in new real-world situations.

The SGs were based on cognitive behavioral theory, social and emotional learning, positive psychology, stress-coping theory, or social cognitive theory; or they incorporated elements from several of these psychological theories. However, for 7 (41%) of the 17 SGs, no theoretical psychological foundation was reported. Most of the studies (15/17, 88%) described the sources used to inform the content design of the intervention. This included (1) input from existing in-person intervention manuals [23,39,44,53,59,61], (2) (systematic) literature reviews [36,57,59,60], (3) input and feedback from target group representatives (eg, via focus groups, codevelopment workshops, and usability tests) [52,56,57,60,61,63,64], and (4) input from experts and other stakeholders (eg, via needs assessments or codevelopment sessions) [42,52,59,61,63].

Of the 17 identified SGs, 10 (59%) were designed as stand-alone interventions, while the gameplay of the remaining games was embedded in broader pedagogical scenarios. These included (1) combining gameplay and classroom sessions throughout the intervention period (*Aislados* [40], *EmoTIC* [49], and *The Adventures of DoReMiFa* [52]), (2) conducting face-to-face group sessions to further discuss the topics addressed in the game after a period of gameplay (*IMPeTUs* [57] and *R.E.M.I.* [63]), (3) inviting people with lived experience of psychological disorders (*Moving Stories* [43,58,59]), and (4) offering individual face-to-face meetings or telephone calls with a counselor (*POD Adventures* [61]).

**Table 2:** Learning objectives of the identified serious games based on the mental health literacy dimensions proposed by Kutcher et al [15].

Serious games	Mental health literacy dimensions [15]			
	Understand how to obtain and maintain good mental health	Understand mental disorders and their treatments	Decrease stigma related to mental disorders	Enhance help-seeking efficacy^a^
Adventures Aboard the S.S. Grin [39]	✓			
Aislados [40]	✓	✓		✓
Emodiscovery [38,42]	✓			✓
EmoTIC [49,56]	✓			✓
Happy 8-12 and Happy 12-16 [34-37]	✓			✓
IMPeTUs [50,57]	✓	✓	✓	✓
Moving Stories [43,58,59]		✓	✓	✓
Unnamed [60]	✓			✓
POD Adventures [46,47,61,62]	✓			✓
Professor Gooley and the Flame of Mind [48]	✓			✓
R.E.M.I. [63]	✓			✓
RegnaTales [44]	✓			✓
REThink [23,29-33,51]	✓			✓
The Singularities [45,64]	✓			✓
Stigma-Stop [22,41]		✓	✓	
The Adventures of DoReMiFa [52]	✓			✓
Zoo U [53]	✓			✓

^a^Knowing when, where, and how to obtain good mental health care and developing competencies needed for self-care.

#### Mechanics

Most of the studies (16/17, 94%) reported on the learning mechanics implemented to achieve the learning objectives. These mechanics can be classified into four broad categories. (1) In many of the SGs (6/17, 35%), players were required to identify and analyze the emotional states and behaviors of nonplayer characters (eg, recognizing basic and complex emotions, distinguishing rational from irrational thoughts, and identifying stressors based on the avatars’ verbal expressions, behaviors, physical appearance, and circumstances). (2) In almost all SGs (14/17, 82%), the player had to propose coping strategies to the nonplayer characters to help them overcome or regulate negative and stressful emotions and irrational thoughts, as well as solve problems, reduce symptoms of mental health disorders, and increase help-seeking behavior. (3) In a few SGs (3/17, 18%), learning was facilitated by presenting psychoeducational content about mental health problems and support strategies. (4) In some SGs (8/17, 47%), the learning objectives were addressed by encouraging players to reflect on their own mental health states and directly practice coping strategies (eg, distraction and breathing techniques as well as journaling) through in-game activities.

The game mechanics included classic point-and-click interactions (where players choose from various response options presented in a virtual menu or conversation window, eg, to select different emotions or propose self-help strategies to nonplayer characters), exploration and observation of the game environment, self-reflective questions and monitoring tools (to support reflection on one’s own mental health states and behavior), and reading assignments (for psychoeducation). Moreover, some minigames featuring activities such as quizzes, flying, running, jumping, collecting objects, painting, shooting, and memory challenges were implemented. Breathing and muscle relaxation techniques were also practiced using minigames.

In addition, minigames and quizzes were mentioned as game elements that enhanced user engagement by providing variety, including cliffhangers (*Adventures Aboard the S.S. Grin* [39]) and self-selection of the sequence in which the game scenarios were played (*Stigma-Stop* [22]). Another important feature for enhancing engagement was the inclusion of individualized feedback based on the player’s in-game performance. This feedback was delivered in the form of (1) points, stars, or virtual money, some of which could be used to upgrade the player’s avatar or purchase new items; (2) direct feedback (written, verbal, or visual) provided by the nonplayer characters; and (3) overall feedback at the end of gameplay regarding helpful and unhelpful behaviors.

Most of the game descriptions were vague regarding how the game progressed based on the player’s in-game behavior. Some of the studies (7/17, 41%) reported that the game proceeded when the player successfully completed the previous module or game episode or reached a defined number of points reflecting their performance. Other studies (4/17, 24%) reported that levels were unlocked after a predefined period (eg, daily or weekly) regardless of the player’s performance on previous levels. Of the 17 studies, 2 (12%) reported that the players’ in-game decisions had an impact on the game’s storyline (*IMPeTUs* [57] and *R.E.M.I.* [63]).

#### Game Design

Of the 17 SGs, 7 (41%) were based on real-world scenarios and included scenes set in a virtual school, home, or playground environment; 7 (41%) used fictional scenarios (mainly nautical or space themed) in which the action takes place on a planet, a sailing ship, or a virtual island; and 3 (18%) used a combination of real-world scenarios and fictional elements. Regarding the narrative and overall game goals, 12 (71%) of the 17 SGs used an overarching storyline in which the game scenarios were embedded. In *REThink*, for example, the main goal is to help people on a fictional planet escape the negative influence of an avatar called the “Irrationalizer” [33]. In *Professor Gooley and the Flame of Mind*, the goal is to find components and activate the “flame of mind” [48]. In *Moving Stories,* the player must improve their relationship with a depressed nonplayer character called Lisa and motivate her to talk to an adult about her problems [59]. In *R.E.M.I.,* the objective is to train a robot to make prosocial decisions [63]. In the remaining SGs, various (social) conflict situations [35,50,56], vignette-based stories [61], or even distinct mobile apps [44] were presented, none of which were interconnected via an overarching storyline.

Most of the SGs (14/17, 82%) were at least partly structured using levels, episodes, modules or game scenes. The recommended playing time varied considerably. In a few of the SGs (3/17, 18%), completing 1 level per week was recommended, while the expected duration per level ranged from 25 to 120 minutes. Among the studies that explicitly reported the intended duration of gameplay, the durations ranged from a single session lasting a few hours (*Emodiscovery*, *Stigma-Stop*, and *R.E.M.I*.) to approximately 1 week (*Moving Stories*), a few weeks (*Adventures Aboard the S.S. Grin*, *POD Adventures*, *Professor Gooley and the Flame of Mind*, *REThink*, and *Zoo U*), and several months (*Aislados*, *EmoTIC*, and *The Adventures of DoReMiFa*). In some of the SGs (5/17, 29%), no specific duration was prescribed, and players were instructed to play at their own pace.

All identified SGs were single-player games. Of the 17 SGs, 5 (29%) were designed to run on PCs only, another 5 (29%) were designed exclusively for mobile apps (smartphone and tablet), and 5 (29%) were compatible with multiple devices, while for 2 (12%) SGs, this information was not reported. Some studies (10/17, 59%) reported information on game features used to enhance realism. Some of the studies (7/17, 41%) indicated that their game scenarios were closely based on real-life experiences and conflicts relevant to the target group [36,37,42,57,59,61,63], which can be regarded as a factor enhancing the SG’s cognitive fidelity. Elements relevant for enhancing the SG’s audiovisual fidelity included voice-overs [39,40,61], background music [40,48], and game environments modeled on real-life school settings and other familiar locations [61]. In addition, players could customize their character based on personal preferences, including gender, name, skin color, outfit, level of education, and body type. Of note, only 1 (6%) of the 17 SGs (*Stigma-Stop* [22]) included an artificial intelligence (AI) component, which was used to generate background and vehicle movement.

#### Results Related to User Engagement as Well as Efficacy and Effectiveness

This section only refers to studies having evaluated the user engagement and efficacy or effectiveness of the included SGs (Table 3). The included studies varied widely in the outcome variables used to evaluate the efficacy and effectiveness of SGs, ranging from emotional outcomes (eg, emotion recognition, emotional intelligence, emotion regulation skills, and empathy) and psychopathology (eg, symptoms of depression, anxiety, and aggression) to life satisfaction and self-esteem. Only a few of the studies (7/17, 41%) included direct measures of mental health literacy, including stigma toward psychiatric disorders [43,59], help-seeking intentions and behavior [43,45,59,64], mental health knowledge [52], or mental health literacy in general [50,57]. Of note, although most of the studies (23/27, 85%) included a few outcome variables, only some (8/27, 30%) explicitly defined a primary outcome variable. The evaluation was based on self-report questionnaires in the great majority of studies (24/27, 89%), with only a few (4/27, 15%) also looking at in-game performance [30,38,42,51]. Of the 27 studies, 2 (7%) used qualitative methodology to evaluate the intervention, including analyzing topics discussed during a classroom session after SG play [58] and assessing users’ satisfaction with the SG and their suggestions for improvement [61]. Pacella and López-Pérez [42] and López-Pérez and Pacella [38] analyzed in-game performance related to correctly identifying emotions and choosing adaptive emotion regulation strategies.

A small number of studies (8/27, 30%) included a follow-up assessment (ranging from 1 to 6 mo), whereas most of the studies (14/27, 52%) used only pretest-posttest assessments. The remaining studies (5/27, 19%) did not include pre-post measures but analyzed in-game performance or usability or acceptability at one time point only.

**Table 3:** Main outcomes related to efficacy and effectiveness, adherence, and dropout in the included evaluation studies.

Serious games and outcome variables (assessment time points)				Main statistical test or analysis method used		Main effects and results		Adherence		Dropout (%)
**Adventures Aboard the S.S. Grin**										
	**Sanchez et al [39]**									
		Social literacy, self-efficacy, social satisfaction, social anxiety, bullying exposure and perpetration (pretest and posttest assessments, with an interval of 9 wk)	Mixed-design ANOVA (time×group interaction)		Significant improvements in social literacy, social satisfaction, social anxiety, and bullying exposure in the IG^a^ compared to the CG^b^ (all *P* values <.05)		74% completed all game episodes		NR^c^	
**Aislados**										
	**Cejudo et al [40]**									
		Quality of life, life satisfaction, positive and negative affect, general mental health, and emotional intelligence (pretest and posttest assessments, with an interval of approximately 6 mo)	MANCOVA^d^ (group differences in posttest scores controlled for pretest scores across outcome variables)		The IG significantly improved across all outcome variables compared to the CG (*P*=.004); effects observed for quality of life, positive affect, and general mental health		NR		NR	
**Emodiscovery**										
	**Pacella and López-Pérez [42]**									
		In-game performance regarding emotion recognition and the use of emotion regulation strategies (N/A^e^)	Descriptive analysis (%)		78%, 67%, and 69% correctly identified sadness, anger, and fear, respectively; most children chose adaptive emotion regulation strategies; children from the United Kingdom less accurate regarding the recognition of sadness than those from Spain; age and gender differences observed regarding the use of adaptive emotion regulation strategies (depending on specific emotion)		NR		NR	
	**López-Pérez and Pacella [38]**									
		In-game performance regarding emotion recognition and the use of emotion regulation strategies (N/A)	Chi-square tests to analyze differences between country, gender, and age		Children from the United Kingdom less accurate regarding the recognition of sadness than those from Spain; age and gender differences observed regarding the use of adaptive emotion regulation strategies (depending on specific emotion)		NR		NR	
**EmoTIC**										
	**de la Barrera et al [49]**									
		Emotional intelligence (PO^f^); self-esteem; affect balance; emotional, behavioral, and peer difficulties; prosocial behavior; depression; anxiety; and stress (pretest and posttest assessments, with an interval of 3 mo)	MANCOVA (group differences in posttest scores controlled for pretest scores across outcome variables)		The IG significantly improved across all outcome variables compared to the CG (*P*=.04); improvement observed for self-esteem, affect balance, emotional symptoms, behavioral problems, and hyperactivity		NR		IG: 71.4; CG: 49.1	
**Happy 8-12 and Happy 12-16**										
	**Filella et al [36]^g^**									
		Emotional development, anxiety, classroom and playground climate, social conflicts, and academic performance (pretest and posttest assessments, with an interval of approximately 1 y)	Mixed-design ANOVA (time×group interaction)		Significant improvements in emotional development (*P*=.02) and anxiety levels (*P*<.001) in the IG compared to the CG; ameliorated playground and classroom atmosphere, a reduction in playground conflicts, and improvements in academic achievement (Spanish grades) observed in the IG compared to the CG		NR		NR	
	**Filella et al [37]^g^**									
		Emotional development, anxiety, classroom and playground climate, social conflicts, and academic performance (pretest and posttest assessments, with an interval of approximately 1 y)	GLM^h^ (time×group interaction)		Significant improvements in the IG compared to the CG regarding emotional awareness (*P*=.04), autonomy (*P*=.03), life competences (*P*=.005), and academic achievements (*P*<.001)		NR		NR	
**IMPeTUs**										
	**Brooks et al [50]**									
		Mental health literacy, quality of life, anxiety, depression, family communication, usability, and acceptability (pretest and posttest assessments, with an interval of 1 mo; 6-mo follow-up, along with qualitative interviews)	Descriptive analysis (means and medians); qualitative interviews		High levels of usability and acceptability regarding interface, personalization, message presentation, and navigation; minimal pretest-posttest changes regarding quantitative outcomes (no significance tests or effect sizes reported)		Mean number of times participants engaged with the intervention: 5 (range 1-15)		9	
**Moving Stories**										
	**Gijzen et al [58]**									
		Implementation costs and themes discussed in debriefing sessions (N/A)	Descriptive analysis (total costs), and qualitative analysis of session notes		Total costs for preparation and offering the intervention: €11,043 (EUR €1=US $1.13); themes discussed (selection): the recognition of depression, help-seeking opportunities in schools, and association between physical and mental health		16.9% did not perform any action; mean 3.1 out of 5 d played; 83% played the game on ≥3 d; gameplay on d 1-5: 48% to 71%		NR	
	**Tuijnman et al [43]**									
		Symptom recognition, first aid confidence, intentions and behavior, beliefs about help, stigma, help-seeking intentions and behavior, and depressive symptoms (pretest and posttest assessments, with an interval of 5 d; 3-mo and 6-mo follow-ups)	Linear mixed effects model (with random intercepts for participants and clusters)		No significant effects for symptom recognition, first aid confidence, intentions and behavior, beliefs about help, help-seeking intentions and behavior, and depressive symptoms in the IG compared to the CG (*P* values: NR); significant reduction in stigma in the IG compared to the CG (*P*=.04); adverse effect: decrease in first aid confidence in the IG from baseline to 6-mo follow-up (*P*<.001)		98% and 94% participated in the introduction and contact sessions, respectively; 49% played the game for 5d, 34% for 4d, 14% for 3d, 3% for 2d or less		Posttest assessment—IG: 1; CG: 2; 6-mo follow-up—IG: 13; CG: 7	
**POD Adventures**										
	**Gonsalves et al [61]**									
		Acceptability and usability (N/A)	Focus groups, co-design workshops, and usability tests		Diverse suggestions to adapt and improve the intervention (eg, blended format, supporting counselors, simplification of language, and interactive elements)		NR		NR	
	**Gonsalves et al [46]**									
		Psychological problem severity, mental health symptoms, perceived stress, and well-being (pretest and posttest assessments, with an interval of 4 wk; 12-wk follow-up)	*t* tests for paired samples (pretest-posttest changes)		Significant improvements in psychological problem severity, mental health symptoms, perceived stress, and well-being, which were maintained at the 12-wk follow-up (all *P* values <.001) in individuals playing the game		Completion of all 4 sessions: 92.7%		Posttest assessment: 32.7; 12-wk follow-up: 52.8	
	**Gonsalves et al [47]**									
		Psychological problem severity, mental health symptoms, perceived stress, and well-being (pretest and posttest assessments, with an interval of 6 wk)	No tests used		No effects reported due to 100% dropout in the IG		80% watched orientation video, 60% completed onboarding session, 20% completed the first game session, and 0% completed sessions 2-4		IG: 100; CG: 33	
**Professor Gooley and the Flame of Mind**										
	**Huen et al [48]**									
		Mental health and psychological well-being (PO), automatic thoughts, self-esteem, procrastination, hope, communication skills, gratitude, problem-solving skills (pretest and posttest assessments, with an interval of 12 wk, before and after each module)	Structural equation model (testing the effect of engagement and attainment on well-being)		The extent of engagement in module activities positively predicted user attainment on psychological constructs; higher attainment on psychological constructs predicted higher psychological well-being after the program (most path coefficients met the threshold for significance)		Cumulative attrition rates after module 1—for modules 2-8, respectively: 37%, 47%, 50%, 53%, 56%, 59%, and 61%		NR	
**RegnaTales**										
	**Ong et al [44]**									
		Aggression levels (pretest and posttest assessments, with an interval of 1 h)	Wilcoxon signed rank test (pretest-posttest changes)		Significant reduction in reactive aggression after playing 3 subgames (*P*=.001, *P*=.008, and *P*=.03); significant reduction in overall aggression after playing 2 subgames (*P*=.008 and *P*=.03); no changes in proactive aggression		NR		NR	
**REThink**										
	**David et al [51]**									
		In-game collected points indicating the identification of functional emotions (N/A)	Friedman test (changes in game performance between game trials)		Significant increase in the ability to correctly identify functional emotions from trial 1 to trial 3 (*P*=.001)		NR		NR	

	**David et al [33]**									
		Emotional symptoms (PO), depressive mood (PO), emotion regulation (emotional awareness and emotional control), the total level of psychological difficulties, conduct problems, hyperactivity, attention, peer problems, and prosocial behavior (pretest and posttest assessments, with an interval of 1 h; 1-mo follow-up)	Mixed within-between MANOVA^i^ and ANCOVAs^j^ for each outcome measure (time×group interaction)		Significant reduction in overall symptoms and difficulties in the IG compared to the CG (*P*=.001); significant decrease in general emotional symptoms (*P*=.002) and depressive mood in the IG (*P*<.001); and significant increase in emotional awareness (*P*<.001) and ability for emotional control (*P*<.001) in the IG		NR		Posttest assessment: 13.94	

	**David et al [29]**									
		Emotional symptoms (PO), depressive mood (PO), emotion regulation (emotional awareness and emotional control), the total level of psychological difficulties, conduct problems, hyperactivity, attention, peer problems, and prosocial behavior (pretest and posttest assessments, with an interval of 1 h; 1-mo follow-up)	Mediation analysis (exploring the mechanisms of change in the IG)		Decrease in irrational beliefs significantly mediated changes in depressive mood (*P*<.001) and overall negative emotions (*P*=.005)		NR		NR	

	**David et al [23]**									
		Emotional problems (PO), cognitive changes (irrational beliefs and negative automatic thoughts), and problem-solving abilities (pretest and posttest assessments, with an interval of 3 wk)	*t* tests for paired samples (pretest-posttest changes)		Significant reduction in emotional problems (*P*=.02) and irrational beliefs (*P*=.02); no significant improvements in automatic thoughts (*P*=.08), rational beliefs (*P*=.20), and problem-solving abilities (*P*=.75)		NR		NR	

	**David et al [31]**									
		Subjective and physiological states of anxiety (pretest and posttest assessments, with an interval of 1 mo; 6-mo follow-up)	Repeated measures ANOVA (time×phase of task×treatment group); mixed models used to analyze alpha asymmetry		Significant decrease in state anxiety (*P*<.001) across groups; no time×group interaction effect; increase in left asymmetry for the serious game IG compared to the face-to-face IG (time×group interaction: *P*<.001)		NR		Posttest assessment—IG: 12.9; face-to-face IG: 18.2; CG: 23.2; 6 mo follow-up—IG: 21.4; face-to-face IG: 34.5; CG: 41.1	
	**David et al [30]**									
		In-game performance, emotional problems, emotion regulation, irrational beliefs, functional and dysfunctional emotions, and problem-solving (pretest and posttest assessments, with an interval of 1 mo)	Correlation analysis		Higher in-game performance at some game levels significantly correlated with improvements in real-world psychological functioning assessed via self-questionnaires (eg, total symptoms: *P*=.03 and frustration tolerance: *P*=.03)		NR		NR	
	**David and Magurean [32]**									
		Emotional problems, functional and dysfunctional emotions, and in-game reaction times for an attentional bias task (pretest and posttest assessments, with an interval of 1 mo)	Correlation analysis		Increase in attentional bias toward positive faces presented in the game was associated with improvements in conduct problems (*P*<.01), hyperactivity (*P*=.02), and peer relationships (*P*=.01), but not with changes in functional and dysfunctional emotions (*P*=.07)		NR		NR	
**The Singularities**										
	**Egan et al [45]**									
		Success of implementation procedures (PO), acceptability (PO), help-seeking intentions, and self-efficacy; coping and flexibility; knowledge and use of web-based resources; cyberbullying exposure, loneliness, mental health, substance use, and internalized stigma (pretest assessment and 1-mo and 2-mo follow-ups)	Descriptive statistics and GLMM^k^ (time×group interaction effect)		High positive affect, low negative affect, low tension and annoyance, and high competence while playing the game (based on benchmarks); significantly larger reductions in cyberbullying exposure (*P*=.05) and binge drinking (*P*=.02) as well as marijuana use frequency (*P*<.01) in the IG compared to the CG		55.8% actually played the game, while 68.2% played the game ≥1 h		1-mo follow-up—IG: 39.2; CG: 25; 2-mo follow-up—IG: 39.2; CG: 32.5	
**Stigma-Stop**										
	**Cangas et al [22]**									
		Attitudes toward schizophrenia (dangerousness and stereotypes; PO; pretest and posttest assessments, with an interval of 3 mo)	*t* tests for paired samples (pretest-posttest changes separately by group)		Significant reduction in negative attitudes toward schizophrenia in the IG (*P*<.001); no changes observed in the CG (*P*=.45)		NR		0	
	**Cangas et al [41]**									
		Attitudes toward schizophrenia (social distance and stereotypes; PO; pretest and posttest assessments, with an interval of 1 h)	*t* tests for paired samples (pretest-posttest changes separately by group)		Significant reduction in negative attitudes toward schizophrenia in the IG (*P*<.01); no changes observed in the CG (*P*=.95); slightly higher effect for high school vs university students		NR		0	
**The Adventures of DoReMiFa**										
	**Shum et al [52]**									
		Anxiety (PO), mental health knowledge, positive and negative thinking, perspective taking, and self-esteem (pretest and posttest assessments, with an interval of 4-6 mo; 6-mo follow-up)	Multilevel models (time×group interaction)		Significant increase in mental health knowledge in the IG compared to the CG after the intervention (*P*=.01) and at 6-mo follow-up (*P*<.001); significant improvements in perspective taking in the IG compared to the CG at 6-mo follow-up (*P*=.03); no effects for anxiety, positive and negative automatic thoughts, and self-esteem (*P* values: NR)		68.9% completed ≥50% of the web-based modules		Posttest assessment—IG: 5.7; CG: 21.5; 6-mo follow-up—IG: 16.3; CG: 42.1	
**Zoo U**										
	**Craig et al [53]**									
		Social skills and literacy, including impulse control, communication, cooperation, social initiation, empathy, emotion regulation, assertiveness skills, and internalizing and externalizing behavior problems; self-efficacy; and feelings of loneliness (pretest and posttest assessments, with an interval of 10 wk)	ANCOVA (group differences in posttest scores controlled for pretest scores)		Significant improvements in parent-reported outcomes in the IG compared to the CG regarding social skills (*P*<.05), including impulse control, emotion regulation, social initiation, assertiveness skills, and externalizing problems; worsening of internalizing behavior problems in the IG (*P*<.05); significant improvements in children-reported outcomes in the IG compared to the CG regarding social self-efficacy, social satisfaction, and social literacy (*P*<.05)		Entire gameplay: mean 8.8 (SD 7.5) h		20	


^a^IG: intervention group.

^b^CG: control group.

^c^NR: not reported.

^d^MANCOVA: multivariate analysis of covariance.

^e^N/A: not applicable.

^f^PO: primary outcome.

^g^The studies by Filella and Ros-Morente [34] and Ros-Morente et al [35] are not presented in this table because they merely repeat the results already reported here.

^h^GLM: generalized linear model.

^i^MANOVA: multivariate analysis of variance.

^j^ANCOVA: analysis of covariance.

^k^GLMM: generalized linear mixed model.

Of the 27 evaluation studies, 10 (37%) reported at least 1 measure of intervention adherence, including the number or percentage of completed game sessions, cumulative attrition rates over the course of game sessions, the number of times the SG was played, or the average duration of gameplay; for example, Sanchez et al [39] and Gonsalves et al [46] reported that 74% and 92.7% of the participants, respectively, completed all game episodes, while Gijzen et al [58] and Egan et al [45] reported the percentage of participants—16.9% and 55.8%, respectively—who never accessed the assigned intervention (although informed consent for the study had been provided). Gonsalves et al [47] reported that all participants discontinued gameplay after the first session. Brooks et al [50] found high usability and acceptability regarding different game components. With regard to assessment dropout, only 12 (44%) of the 27 studies reported relevant data, with posttest dropout rates in the intervention groups ranging from 0% to 100% (median 13.4%, IQR 2.2%-37.6%). Regarding the efficacy of the SGs, studies that used an RCT or quasi-experimental design reported significantly greater improvements in at least 1 outcome variable compared to a waitlist or active control condition, except for Gonsalves et al [47], who were not able to analyze the data due to a 100% dropout rate in the intervention group (Table 3). In studies that included a follow-up assessment, at least some of the effects were maintained in the longer term. The study by Tuijnman et al [43] explicitly reported a potential adverse effect: first aid confidence significantly decreased in the intervention group compared to the control group at the final follow-up (Table 3). The study by Gijzen et al [58] was the only one that calculated the implementation costs of the intervention.

### Quality Appraisal of the Included Studies

The results of the quality appraisal of the controlled intervention studies and uncontrolled pretest-posttest evaluation studies are shown in Tables S3 and S4 in Multimedia Appendix 2. Of the 15 controlled intervention studies, only 6 (40%) met more than half of the predefined quality criteria, while no study met >9 out of 14 quality criteria. None of the uncontrolled pretest-posttest evaluation studies met >5 out of 12 assessed quality criteria. In general, a relatively high percentage of quality criteria (approximately 22%) could not be assessed because the papers did not report the required information. This indicates that the overall quality of the included studies was relatively low.

### Fostering and Hindering Factors Based on the RE-AIM Framework

With respect to participant reach, two factors were repeatedly discussed in the included studies. First, ease of access: cost-free access to the digital game, availability across different devices and operating systems, and a simple and intuitive interface were reported to facilitate participant reach [22,38,42,50,51,56,63]. By contrast, compatibility problems between devices [31,64], general technical issues when downloading the game, and poor internet connectivity [47,50] were mentioned as potential hindering factors. Second, availability in different languages: some of the studies (6/36, 17%) pointed to the importance of offering the SG in different (local) languages [40,46,47,60-62].

Additional factors discussed in the studies included availability of the SG through a trustworthy organization, familiarity with digital games in general [50], and introduction of the intervention by a facilitator or counselor [46,47,62], all of which were cited as fostering factors for participant reach. Parental concerns regarding digital games for children were mentioned as a hindering factor in the study by Brooks et al [50].

Several factors potentially influencing the efficacy and effectiveness of SGs, as well as user engagement, were discussed in the included studies. Game design elements potentially fostering engagement and effectiveness included an appealing narrative [39,50], a simple and intuitive interface [42], and game characters who were the same age as the target group [38]. By contrast, an intervention period that was too short [23,31,32,35-37,52] or too long [50], poor graphics, the absence of voice-overs [42], excessive text [50], and repeated game crashes [45] were considered hindering factors. Regarding game mechanics, self-customizable characters [39,45] and feedback from nonplayable characters [63] were reported to potentially increase efficacy or effectiveness. By contrast, long waiting times to access the next game level and being trapped in a game level until it was successfully completed were identified as factors that could potentially negatively impact engagement [48]. In addition, regular reminders to use the game were reported to improve adherence [46,47]. Relevant implementation aspects potentially fostering effectiveness included the involvement of teachers or other counselors as facilitators [34,46,48,52,62]; group discussion sessions about the game content [50,52], particularly when facilitated by people with lived experience of mental health problems [22,43]; and the involvement of parents in gameplay [50]. Two studies emphasized that older children may profit more from an SG than younger children [43,50]. Gijzen et al [58] reported that group dynamics have a positive effect on adherence, in the sense that as more students play the game, discussion about the game content increases, which in turn leads to better adherence.

No fostering or hindering factors related to adoption (ie, whether organizations such as schools were willing to test or initiate a program) were reported in the included studies.

Regarding fostering factors related to the implementation of SGs in a specific setting (most commonly schools), the most frequently discussed factor was the involvement of, and training for, facilitators, including teachers, counselors, and workers with lived experience [40,43,50,58]. Furthermore, the suitability of the intervention for implementation in classrooms [36] and the availability of support for technical problems and questions about the game content [47,62] were identified as potentially beneficial for implementation. Time pressure and the competing responsibilities of facilitators (eg, teachers) were considered hindering factors for implementation [50].

To foster the maintenance of effects, the option to expand the game through additional modules was discussed [50]. With regard to intervention sustainability, embedding the SG within a more general educational context and the school curriculum [40,52,58,60] as well as ensuring cost-free access to the SG across different platforms were emphasized [22,42,52].

## Discussion

### Principal Findings

The aim of this study was to systematically review SGs designed to promote mental health literacy among adolescents aged 10 to 14 years, with a focus on game characteristics, efficacy and effectiveness, and factors contributing to implementation success. We identified 36 articles that described the development of 17 SGs and evaluated their feasibility, efficacy, and effectiveness. The specific learning objectives of the identified SGs varied considerably across the included studies. Many SGs focused on the promotion of general aspects of mental health (eg, improving skills to regulate emotion and stress), thus reflecting the components of mental health literacy as conceptualized by Kutcher et al [15], including the promotion of good mental health and the development of skills for self-care [15], while classic aspects of mental health literacy, such as increasing knowledge as well as improving the recognition of specific psychiatric disorders and reducing stigma [14], were addressed by only a few of the studies. By contrast, another review of non–game-based mental health literacy interventions for adolescents reported that increasing knowledge of psychiatric disorders and reducing stigma were the most common learning objectives [17]. This may be explained by the fact that our review focused on SGs designed for a younger age group, but it could also indicate that a game-based approach can be particularly useful for promoting specific mental health literacy skills, while knowledge about specific psychiatric disorders may be better communicated via offline interventions. Of note, promoting overarching aspects of mental health such as emotion regulation rather than focusing on specific aspects or symptoms of mental health have been previously identified as particularly useful for preventive interventions in adolescents [65].

Many of the studies included in this review used a co-design approach to develop the SG, involving target group representatives, mental health experts, software developers, and other relevant stakeholders (eg, school personnel). It has been argued that user involvement should be standard practice when developing digital mental health interventions because this is essential for ensuring user engagement and intervention effectiveness [66]. User and stakeholder involvement is recommended at all stages of the development process, including initial planning, early design testing, prototyping, feasibility testing, and implementation [67,68]. In-depth stakeholder involvement during game development seems crucial: many of the SGs identified in this review were implemented in a school setting and thus embedded within a larger pedagogical scenario. As repeatedly discussed in the included studies, teachers are often regarded as important facilitators when digital mental health interventions are implemented in schools; therefore, facilitator training seems important for ensuring intervention success [69]. In addition to information about the structure and content of the SG, facilitator training should also incorporate guidance on how an SG can be best introduced in the classroom context and how to promote discussions about the game content with students. Indeed, face-to-face group discussions about the game content have been discussed as a factor enhancing engagement during gameplay as well as improving outcomes [57]. The involvement of individuals (previously) affected by psychiatric disorders may further facilitate discussions among users, enhance the credibility of the game content, and improve the sustainability of effects [43,52,59]. By contrast, the absence of human support was mentioned as one of the main reasons for the high attrition rate in an SG [48]. Blended interventions have been previously discussed as promising with regard to user engagement and efficacy [70], which supports the findings of this review. Moreover, this study also suggests that researchers should consider early how a game-based intervention can be integrated into the school curriculum; this was mentioned as a key point for intervention sustainability in some of the included studies [57,61], and it has also been emphasized as an important factor in a previous systematic review of web-based mental health interventions [71] and in a stakeholder survey among school professionals [69].

Regarding game mechanics, this review shows that the identified SGs used a large variety of mechanics, including selecting response options when interacting with nonplayer characters, jumping, running, flying, collecting objects, painting, shooting, and freely exploring the game environment. Many of the identified SGs combined different mechanics. The use of diverse game mechanics may represent an important aspect for user experience and user engagement because previous research has shown that different types of players exhibit varying motivations for playing and have different preferred interaction styles [72,73]; for example, the “socializer” player type may be particularly engaged by interacting with other players or nonplayer characters, while “free spirits” may be motivated by tasks associated with autonomy and self-expression, which could be addressed by the possibility to explore the game environment (such as in open-world games). By contrast, “killers” are motivated by competitive game elements, such as leaderboards and rankings. With regard to the future development of SGs in the mental health field, incorporating several different game mechanics seems crucial to fit the players’ needs and preferences. This seems particularly relevant, considering that game mechanics tailored to the users’ needs may facilitate “flow” during gameplay—a mental state in which gamers become so engrossed in the game that they lose sense of time [74] and which has been associated with user engagement in gameplay [75,76]. Moreover, in the positive emotion, engagement, relationships, meaning, and accomplishment model formulated by Seligman [77], engagement, defined as the ability to fully use one’s strengths and abilities, is seen as a core component of psychosocial well-being; therefore, game mechanics that support individual strengths and abilities may foster the feeling of flow during gameplay.

Many of the SGs identified in this review allowed some form of customization of the player’s in-game character. This feature was discussed as potentially fostering intervention effectiveness [39,45]. Indeed, avatar customization facilitates identification with the player’s character [78] and has been associated with the players’ perceived competence in the game, the level of perceived fun, and self-reported game performance [79]. Customization seems particularly important for users with special needs, such as sexual and gender minority youth, because character customization and game character diversity can help to better reflect who they feel they are or want to be and give them an opportunity to rehearse their gender identity [80,81].

Another important aspect for fostering identification and engagement in the SGs discussed in the included studies is the chosen game universe and storyline. We identified a mix of SGs set in fictional worlds or worlds resembling real life. A previous study reported that adolescents generally prefer realistic settings (eg, public places and cities) over fictional settings; however, gender differences should also be taken into account because fictional worlds and nonhuman characters were found to be more appealing to boys [82]. This indicates that customization should not be limited to the player’s character, as discussed previously; it should also be considered for the narrative and storyline. It could be argued that game settings reflecting adolescents’ real-world environments may facilitate the transfer of mental health literacy–related skills learned through the SG to everyday life; however, further research is needed.

Of note, AI components were used in only 1 (6%) of the 17 SGs identified in our review (to generate background movement in the game) [22]. In general, AI has not yet been widely used in SGs targeting mental health, aside from a few therapeutic games addressing specific psychiatric disorders. A recently published review showed that AI was primarily applied for disease detection and the evaluation of user performance in SGs targeting individuals with motor impairment, while only a small number of SGs applied this technology in patients with attention-deficit/hyperactivity disorder or autism or in healthy individuals [83]. Nevertheless, integrating AI components into SGs for player modeling (eg, real-time emotion recognition), natural language processing (eg, the detection of emotional states), and believable nonplayer characters (eg, nonverbal bodily motion) has been discussed as a future direction for SG development [84] and may also be relevant for SGs addressing mental health literacy in adolescents. Moreover, AI could also be used to guide game progression based on a player’s previous in-game behavior.

Another aspect observed in this review was the wide variation in recommended or actual SG play duration, ranging from a single session of approximately 1 hour to a whole school year. For the future development of SGs for adolescents, it would be worth considering whether an optimal duration of gameplay exists to achieve the best outcomes and ensure long-lasting effects. In some of the included studies, a low number of game levels or a relatively brief intervention period (only several weeks in most cases) were discussed as potentially limiting the effects [23,36,37,42]. By contrast, Brooks et al [50] noted that extending the playtime of their game, which lasts only 4 hours, would have reduced users’ motivation to play. Another review that provided guidelines for developing game-based mental health interventions merely stated that a game should be neither too long nor too short [85], without offering specific recommendations. Thus, no definite conclusion about the optimal duration of SG play can be drawn to date, and future studies should address this issue. At present, it can be stated that it seems important that an SG targeting mental health literacy provides sufficiently long gameplay to consolidate effects and incorporates game mechanics that keep users engaged, even when game sequences are repeated.

The included evaluation studies provided some evidence for the short-term efficacy of SGs targeting aspects of mental health literacy in adolescents, thereby supporting the evidence from non–game-based interventions in this age group [12,86]. However, there are some reasons for why these effects should be considered as preliminary: First, the quality appraisal of the included studies revealed that most of them were generally of low quality. Second, many of the studies were pilot studies with small samples or lacked control groups. Third, the great majority of studies did not include follow-up measures to evaluate whether the observed effects were sustained over time. This limitation was also mentioned in other reviews of gamified mental health interventions for children and adolescents [25,87,88]. Fourth, assessments of intervention adherence and dropout were often not included in the evaluation reports. However, the reporting of attrition and use parameters seems particularly important for digital health interventions [89] because these data provide valuable information on user engagement. Notably, some of the studies reported relatively low adherence. Fifth, the evaluation of most of the SGs identified in the review relied exclusively on self-report instruments, which has also been emphasized as a limitation in other reviews [87,88].

With a few exceptions, users’ in-game performance was neither measured nor reported. The evaluation of users’ specific in-game behavior would provide important information on whether they behaved in accordance with the intended objectives and which parts or tools of an SG were used and for how long, which can further be used to evaluate and improve user experience and engagement. Moreover, only 1 study (on the *REThink* intervention [29]) evaluated the potential mechanism of change and found that a reduction in irrational beliefs mediated changes in depressive mood. Furthermore, most of the studies used specific measures closely related to the learning objectives of the SG rather than more general measures of mental health literacy. As outlined by Mansfield et al [90], this may be due to the scarcity of psychometrically validated instruments measuring mental health literacy in adolescents, with existing instruments often based on an illness-focused definition of mental health literacy that often does not incorporate the assessment of skills to obtain positive mental health. However, in view of the increasing interest in improving mental health literacy during adolescence, research on the development of assessment instruments suitable for this age group is emerging [91-93].

The included studies provided limited information on the potential disadvantages of using SGs for promoting mental health literacy. The available data point to potential implementation challenges, with concerns from parents or facilitators (eg, teachers) regarding the use of these technologies being the most important issues (eg, too much screen time in general and doubts about how to integrate an SG intervention into the school context). Fleming et al [24] further suggest that the development of digital games for scientific purposes often cannot compete with those developed for commercial purposes, which typically have much larger budgets. As a result, games developed for scientific purposes may often not be as technologically up to date, potentially influencing users’ engagement levels. Moreover, some users or implementers may perceive the use of a digital game to address mental health issues as inappropriate or trivializing [24], which underlines the need for user and stakeholder involvement at all stages of the game development.

### Strengths and Limitations

This systematic review presents a comprehensive overview of SG characteristics, including learning design, mechanics, and game design, for use in a universal population of adolescents, targeting aspects of mental health literacy. As the coding scheme was closely oriented toward the dimensions of the co.LAB framework [26], a framework used for the collaborative design of SGs, the results from this review can directly inform the design of future SGs in this field. Another strength is that we used the RE-AIM framework, which was also applied in a previous systematic review of digital mental health interventions [71] and in a review of health literacy interventions [94], enabling comparability with the previous results. Moreover, as we focused on SGs appropriate for a relatively narrow age range (10-14 y), this study revealed SG characteristics that may be particularly relevant for this population. A limitation is that, due to the diversity of study designs and outcome measures used in the included studies, we were not able to aggregate the effects using a meta-analytic approach. Moreover, considering the number of RQs addressed in this review, only an overview of the main findings of the included studies regarding efficacy and effectiveness was provided. Furthermore, the literature search was limited to 3 databases (PubMed, Scopus, and PsycINFO) and the reference lists of the included studies. Although this search strategy yielded a comprehensive set of relevant studies, it cannot be ruled out that we have overlooked other relevant studies that could have been retrieved by including additional literature databases (eg, those with a greater focus on technology).

### Conclusions

Overall, this review showed that SGs are a promising approach to improve mental health literacy in adolescents. However, the evidence on the efficacy and effectiveness of SG interventions in this field should be regarded as preliminary. More rigorously planned studies, including RCTs and real-world trials, are needed. These studies should incorporate (long-term) follow-up measures and assessments of in-game performance, in addition to self-reports and measures of intervention adherence. This review also highlights commonly used characteristics of SG design, which can inform the development of future game-based interventions for promoting mental health literacy. A content-related focus on promoting positive mental health and teaching self-management skills, such as emotion regulation and stress-coping skills, which are relevant to nearly all children and adolescents, may be more effective than focusing on mental health literacy related to (symptoms of) specific psychiatric disorders. Intervention co-design approaches and in-depth stakeholder involvement at all stages of the SG development seem crucial for its success. In particular, close collaboration with stakeholders from the school setting (eg, teachers and principals) is of high importance for discussing how an SG can be best embedded within a wider pedagogical scenario and how to facilitate intervention sustainability. The use of different mechanics within a single SG may be useful to fit diverse player needs and preferences, foster a state of flow during gameplay, and increase engagement. Furthermore, customization of the SG to meet the user’s needs, for example, customizing the player’s character as well as the game narrative, may be useful features to strengthen the player’s relatedness to the game characters and content, potentially enhancing the SG’s real-world effectiveness.

## Appendix

Multimedia Appendix 1 PRISMA checklist.

Multimedia Appendix 2 Supplementary tables, including search syntax for PubMed, Scopus and PsycINFO; systematic review coding sheet; quality appraisal of controlled intervention studies; and quality appraisal of uncontrolled pretest-posttest studies.

Multimedia Appendix 3 Coded characteristics of serious games (design elements based on the co.LAB framework).

## Abbreviations

AI: artificial intelligence
PRISMA: Preferred Reporting Items for Systematic Reviews and Meta-Analyses
RCT: randomized controlled trial
RE-AIM: Reach, Effectiveness, Adoption, Implementation, and Maintenance
RQ: research question
SG: serious game
