# Supplementary material for: Game Design, Effectiveness, and Implementation of Serious Games Promoting Aspects of Mental Health Literacy Among Children and Adolescents: Systematic Review
Source: JMIR Ment Health. 2025 May 5;12:e67418. doi: 10.2196/67418 (PMC12089885; doi:10.2196/67418)
Supplement: Multimedia Appendix 2 [file mental_v12i1e67418_app2.docx]

**Supplementary File 1**

**Table S1.** Syntax of the literature search in PubMed, Scopus and PsycInfo

| **PubMed** |
| --- |
| (“mental health” [Title/Abstract] OR "mental health literacy"[Title/Abstract] OR "mental health promotion"[Title/Abstract] OR "coping"[Title/Abstract] OR "stress management"[Title/Abstract] OR "emotion recognition"[Title/Abstract] OR "emotion regulation"[Title/Abstract] OR "stigma"[Title/Abstract]) AND ("serious game*"[Title/Abstract] OR "digital game*"[Title/Abstract] OR "serious video game*"[Title/Abstract] OR "game-based"[Title/Abstract] OR "gamification"[Title/Abstract] OR "smartphone game*"[Title/Abstract] OR "mobile game*"[Title/Abstract]) |
| **Scopus** |
| ( TITLE-ABS-KEY ( “mental health” OR "mental health literacy" OR "mental health promotion" OR coping OR "stress management" OR "emotion recognition" OR "emotion regulation" OR stigma ) AND TITLE-ABS-KEY ( "serious game*" OR "digital game*" OR "serious video game*" OR "game-based" OR gamification OR "smartphone game*" OR "mobile game*" ) ) |
| **PsycInfo** |
| ((mental health OR mental health literacy OR mental health promotion OR coping OR stress management OR emotion recognition OR emotion regulation OR stigma) AND (serious game* OR digital game* OR serious video game* OR game-based OR gamification OR smartphone game* OR mobile game*)).mp |

**Table S2.** Systematic review coding sheet

| **Coding of game design elements based on the co.Lab framework (Jaccard et al., 2021)** | |
| --- | --- |
| **Item** | **Definition**^a^ |
| **Learning Design** | |
| **Learning Profiles** | Profile of the target population |
| **Learning Functions** | Whether the goal of the game is (1) to test or apply existing knowledge and skills, (2) support knowledge or skill acquisition or (3) to prepare for future courses |
| **Learning Objectives** | Definition of what the participants should have learned after playing the game |
| **Learning Foundations** | Underlying learning theories and pedagogical methods to achieve the learning objectives |
| **Knowledge Foundations** | How relevant skills and knowledge is retrieved to design the intervention (e.g. expert interviews, literature review) |
| **Pedagogical Scenario** | General structure of the program (stand-alone intervention vs. blended intervention) |
| **Mechanics** | |
| **Learning Mechanics** | In-game activities such as remembering, understanding, applying, analyzing, evaluating, or creating |
| **Game Mechanics** | Basic game elements of interactivity, set of actions which are done by the player |
| **Learning and Game incentives** | Rewards in the game for supporting participants’ engagement and motivation, can be intrinsic or extrinsic |
| **Learning and Game interactions** | interactions in the game which help with the learning process, includes feedback to the player on their game behavior and how the game proceeds based on the player’s actions |
| **Game Design** | |
| **Goal and rules** | Activities and interaction the player will be allowed to perform (game rules) to achieve the overall objective (game goal) |
| **Game Universe** | World in which the game takes place (e.g. fictional world vs. simulation of the real world) |
| **Fidelity and simulation model** | Features which are used to enhance realism (including audio-visual fidelity, narrative fidelity, cognitive fidelity) |
| **Interfaces and User experience** | Including game elements to increase user experience (e.g. flow), user interface, usability aspects and the devices used |
| **Game Structure** | Description of the game and the learning progression, e.g. including number of levels, intended length and pace |
| **Narrative** | Content and structure of the story |
| **Coding of study and sample characteristics** | |
| **Study characteristics** | |
| **First author** | Name of first author |
| **Publication year** | Year of publication |
| **Country of intervention delivery** |  |
| **Study design** | e.g. RCT, cluster RCT, qualitative study, pilot study, cohort study, cross-sectional study. study protocol |
| **Study arms** | e.g. intervention group vs. waitlist-control group |
| **Target group and sample characteristics** | |
| **Inclusion & Exclusion criteria** | List of defined inclusion and exclusion criteria |
| **Sample size** | Total number of participants (and per study, if applicable) |
| **Participation rate** | Percentage of individuals participating in the study (of those who were approached) |
| **Mean age + SD** | Mean age (+ SD) of participants (per study arm, if applicable) |
| **Age range** | Age range of the sample |
| **% females** | Percentage of female participants (per study arm, if applicable) |
| **Implementation setting** | e.g. schools, at home |
| **Outcomes and results** | |
| **Outcome variable(s)** | Outcome variables used to evaluate the effectiveness of the intervention; e.g. MHL general, emotion regulation, stress coping, stigma, depression |
| **Primary outcome** | Outcome variable defined as the primary outcome |
| **Follow-ups** | Assessment time points to evaluate the intervention |
| **Adherence** | Any measure included to assess intervention adherence + respective results |
| **Dropout out rate** | % of dropout at post-intervention assessment and follow-up assessments (if applicable) |
| **Main effects and results of the study** | Summary of main results and effects of the study regarding the evaluation the serious game intervention |
| **Implementation aspects: Fostering and hindering factors according to the REAIM framework (Glasgow et al., 1999)** | |
| **Fostering & hindering factors for reach** | Fostering and hindering factors for reach of the target population (that a large proportion of individuals are willing to participate in the program) |
| **Fostering & hindering factors for effectiveness** | Fostering and hindering factors for the impact of an intervention on important outcomes and adherence |
| **Fostering & hindering factors for adopting** | Fostering and hindering factors for organizational adoption (that a large proportion of organizations, e.g. schools, are willing to initiate a program) |
| **Fostering & hindering factors for implementation** | Fostering and hindering factors for the implementation of a program in a specific setting, meaning that a program can be implemented, e.g. in the school setting, as intended. |
| **Fostering & hindering factors for maintenance** | Fostering and hindering factors for long-term sustainability of individual effects and long-term sustainability of the program itself. |

**Table S3.** Quality appraisal of controlled intervention studies using the NIH quality assessment tool

|  | Sanchez et al. (2017) | Cejudo et al. (2020) | De la Barrera et al. (2021) | Filella et al. (2016)^a^ | Filella et al. (2018)^a^ | Tuijnman et al. (2022) | Gonsalves et al. (2023) | David et al. (2019a) | David et al. (2019b)^b^ | David et al. (2022b)^b^ | Egan et al. (2021) | Cangas et al. (2017) | Cangas et al. (2019) | Shum et al. (2019) | Craig et al. (2016) |
| --- | --- | --- | --- | --- | --- | --- | --- | --- | --- | --- | --- | --- | --- | --- | --- |
| 1. Study described as RCT | ❶ | ⓿ | ❶ | ⓿ | ⓿ | ❶ | ❶ | ❶ | ❶ | ❶ | ❶ | ⓿ | ⓿ | ⓿ | ⓿ |
| 2. Randomization method adequate | NR | ⓿ | ❶ | ⓿ | ⓿ | ❶ | ❶ | NR | NR | NR | ❶ | NR | ⓿ | ⓿ | NR |
| 3. Treatment allocation concealed | NR | NR | ❶ | ⓿ | ⓿ | ❶ | ❶ | NR | NR | NR | ❶ | NR | ⓿ | ⓿ | NR |
| 4. Participants and providers blinded | ⓿ | ⓿ | ⓿ | ⓿ | ⓿ | ⓿ | ⓿ | ⓿ | ⓿ | ⓿ | ⓿ | ⓿ | ⓿ | ⓿ | ⓿ |
| 5. Outcome assessors blinded | NA | NA | NA | ⓿ | NA | NA | ❶ | ⓿ | ⓿ | NR | ⓿ | NR | ⓿ | NA | NR |
| 6. Groups similar at baseline | ❶ | ❶ | ❶ | NR | NR | ⓿ | ❶ | ❶ | ❶ | ❶ | ❶ | ❶ | NR | ❶ | ❶ |
| 7. Overall dropout rate < 20% | NR | NR | ⓿ | NR | NR | ❶ | ⓿ | ❶ | ❶ | ❶ | ⓿ | NR | NR | ❶ | ❶ |
| 8. Differential drop-out rate <15% | NR | NR | ⓿ | NR | NR | ❶ | ⓿ | ❶ | ❶ | ❶ | ❶ | NR | NR | ⓿ | NR |
| 9. High adherence to intervention protocols | ❶ | NR | NR | NR | NR | ⓿ | ⓿ | NR | NR | NR | ⓿ | NR | NR | ⓿ | ❶ |
| 10. Other interventions similar across groups | NR | ❶ | NR | NR | NR | ❶ | ❶ | NR | NR | NR | NR | NR | NR | NR | NR |
| 11. Valid and reliable outcome measures | ❶ | ❶ | ❶ | ❶ | ❶ | ⓿ | ❶ | ❶ | ❶ | ❶ | ❶ | ❶ | ❶ | ❶ | ❶ |
| 12. Sample size sufficiently large (≥80% power) | ⓿ | ⓿ | ⓿ | ⓿ | ⓿ | ❶ | ⓿ | ❶ | ❶ | ❶ | ⓿ | ⓿ | ⓿ | ⓿ | ⓿ |
| 13. Outcomes/analyses pre-specified | ⓿ | ⓿ | ❶ | ⓿ | ⓿ | ❶ | ❶ | ❶ | ❶ | ❶ | ❶ | ⓿ | ⓿ | ⓿ | ⓿ |
| 14. Randomized participants analyzed in group they were assigned to (ITT analysis) | NR | NR | NR | NR | NR | ❶ | NA | ❶ | ❶ | ❶ | ❶ | NR | NR | ❶ | ⓿ |
| Total points | 4/14 | 3/14 | 6/14 | 1/14 | 1/14 | 9/14 | 8/14 | 8/14 | 8/14 | 8/14 | 8/14 | 2/14 | 1/14 | 4/14 | 4/14 |

**⓿** criterion not met, ❶ criterion met, *NA* not applicable, *NR* not reported
^a^ The studies by Ros-Morente et al. (2018) and Fillela & Ros-Morente et al. (2023) are not included in this table as they just reported a summary of results already published by Fillela et al. (2016) and Fillela et al. (2018).

^b^ These are secondary analyses papers of David et al. (2019a). Some ratings are also based on the information provided in David et al. (2019a).

**Table S4.** Quality appraisal of uncontrolled pre-post evaluation studies using the NIH quality assessment tool

|  | Pacella & López-Pérez (2018) | López-Pérez & Pacella (2021) | Brooks et al. (2023) | Gonsalves et al. (2021b) | Huen et al. (2016) | Ong et al. (2019) | David et al. (2018) | David et al. (2021) | David et al. (2022a)^a^ | David & Magurean (2022)^a^ |
| --- | --- | --- | --- | --- | --- | --- | --- | --- | --- | --- |
| 1. Clearly stated study objectives | ⓿ | ❶ | ❶ | ❶ | ❶ | ❶ | ❶ | ❶ | ❶ | ❶ |
| 2. Clearly described eligibility criteria | ⓿ | ⓿ | ❶ | ❶ | ⓿ | ⓿ | ⓿ | ⓿ | ⓿ | ⓿ |
| 3. Representativeness of participants | ⓿ | ⓿ | ⓿ | ⓿ | NR | ⓿ | ⓿ | ⓿ | NR | NR |
| 4. High participation rate | ❶ | ❶ | NR | ⓿ | ⓿ | NR | ⓿ | ❶ | ⓿ | NR |
| 5. Sample size sufficiently large | ⓿ | ❶ | ⓿ | ⓿ | ❶ | ⓿ | ⓿ | ⓿ | ⓿ | ⓿ |
| 6. Intervention clearly described and delivered consistently | ❶ | ❶ | ❶ | ❶ | ❶ | ❶ | ❶ | ❶ | ❶ | ❶ |
| 7. Valid and reliable outcome measures | ❶ | ❶ | ⓿ | ❶ | ❶ | ❶ | ❶ | ❶ | ❶ | ❶ |
| 8. Outcome assessors blinded | NA | NR | NR | NR | NA | NR | NA | NA | NA | NA |
| 9. Dropout rate < 20% | NR | NA | ❶ | ⓿ | ⓿ | NR | NA | NR | ❶ | ❶ |
| 10. Statistical test used to evaluate pre-post changes | ❶ | ⓿ | ⓿ | ❶ | ⓿ | ❶ | ❶ | ❶ | ❶ | ⓿ |
| 11. Outcomes assessed multiple time before and after intervention | ⓿ | ⓿ | ⓿ | ⓿ | ⓿ | ⓿ | ⓿ | ⓿ | ⓿ | ⓿ |
| 12. Individual-level data analysis (if intervention conducted at group level) | ❶ | NA | NA | NA | NA | NA | NA | NA | NA | NA |
| Total points | 5/12 | 5/12 | 4/12 | 5/12 | 4/12 | 4/12 | 4/12 | 5/12 | 5/12 | 4/12 |

**⓿** criterion not met, ❶ criterion met, *NA* not applicable, *NR* not reported
^a^ These are secondary analyses papers of David et al. (2019a). Some ratings are also based on the information provided in David et al. (2019a).

**References**

Brooks H, Irmansyah I, Syarif AK, et al. Evaluating a prototype digital mental health literacy intervention for children and young people aged 11-15 in Java, Indonesia: a mixed methods, multi-site case study evaluation. *Child Adolesc Psychiatry Ment Health*. 2023;17(1):79. doi:10.1186/s13034-023-00608-9

Cangas AJ, Navarro N, Aguilar-Parra JM, et al. Analysis of the Usefulness of a Serious Game to Raise Awareness about Mental Health Problems in a Sample of High School and University Students: Relationship with Familiarity and Time Spent Playing Video Games. *J Clin Med*. 2019;8(10):1504. doi:10.3390/jcm8101504

Cangas AJ, Navarro N, Parra JMA, et al. Stigma-Stop: A Serious Game against the Stigma toward Mental Health in Educational Settings. *Front Psychol*. 2017;8:1385. doi:10.3389/fpsyg.2017.01385

Cejudo J, Losada L, Feltrero R. Promoting Social and Emotional Learning and Subjective Well-Being: Impact of the “Aislados” Intervention Program in Adolescents. *Int J Environ Res Public Health*. 2020;17(2):609. doi:10.3390/ijerph17020609

Craig AB, Brown ER, Upright J, DeRosier ME. Enhancing Children’s Social Emotional Functioning Through Virtual Game-Based Delivery of Social Skills Training. *J Child Fam Stud*. 2016;25(3):959-968. doi:10.1007/s10826-015-0274-8

David OA, Cardoș RAI, Matu S. Changes in irrational beliefs are responsible for the efficacy of the REThink therapeutic game in preventing emotional disorders in children and adolescents: mechanisms of change analysis of a randomized clinical trial. *Eur Child Adolesc Psychiatry*. 2019b;28(3):307-318. doi:10.1007/s00787-018-1195-z

David OA, Cardoș RAI, Matu S. Is REThink therapeutic game effective in preventing emotional disorders in children and adolescents? Outcomes of a randomized clinical trial. *Eur Child Adolesc Psychiatry*. 2019a;28(1):111-122. doi:10.1007/s00787-018-1192-2

David OA, Magurean S. Positive Attention Bias Trained during the Rethink Therapeutic Online Game and Related Improvements in Children and Adolescents’ Mental Health. *Child Basel Switz*. 2022;9(11):1600. doi:10.3390/children9111600

David OA, Magurean S, Tomoiagă C. Do Improvements in Therapeutic Game-Based Skills Transfer to Real Life Improvements in Children’s Emotion-Regulation Abilities and Mental Health? A Pilot Study That Offers Preliminary Validity of the REThink In-game Performance Scoring. *Front Psychiatry*. 2022a;13:828481. doi:10.3389/fpsyt.2022.828481

David OA, Predatu R, Cardoș RAI. Effectiveness of the REThink therapeutic online video game in promoting mental health in children and adolescents. *Internet Interv*. 2021;25:100391. doi:10.1016/j.invent.2021.100391

David O, Predatu R, Roxana C. A pilot study of the REThink online video game applied for coaching emotional understanding in children and adolescents in the therapeutic video game environment: The Feeling Better resources game. *J Evid-Based Psychother*. 2018;18:57-68. doi:10.24193/jebp.2018.1.5

David OA, Stroian PI, Predatu R, Maffei A. State anxiety and frontal alpha asymmetry effects of the REThink online video game for children and adolescents: A six-month follow-up. *Personal Individ Differ*. 2022b;196. doi:10.1016/j.paid.2022.111725

De la Barrera U, Mónaco E, Postigo-Zegarra S, Gil-Gómez JA, Montoya-Castilla I. EmoTIC: Impact of a game-based social-emotional programme on adolescents. *PloS One*. 2021b;16(4):e0250384. doi:10.1371/journal.pone.0250384

Egan JE, Corey SL, Henderson ER, et al. Feasibility of a Web-Accessible Game-Based Intervention Aimed at Improving Help Seeking and Coping Among Sexual and Gender Minority Youth: Results From a Randomized Controlled Trial. *J Adolesc Health Off Publ Soc Adolesc Med*. 2021;69(4):604-614. doi:10.1016/j.jadohealth.2021.03.027

Filella G, Cabello E, Escoda N, Ros-Morente A. Evaluation of the Emotional Education program “Happy 8-12” for the assertive resolution of conflicts among peers. *Electron J Res Educ Psychol*. 2016;14:582-602. doi:10.14204/ejrep.40.15164

Filella G, Ros-Morente A. Happy Software: An interactive program based on an emotion management model for assertive conflict resolution. *Front Psychol*. 2023;13. doi:10.3389/fpsyg.2022.935726

Filella G, Ros-Morente A, Oriol X, March-Llanes J. The Assertive Resolution of Conflicts in School With a Gamified Emotion Education Program. *Front Psychol*. 2018;9:2353. doi:10.3389/fpsyg.2018.02353

Gonsalves PP, Bhat B, Sharma R, et al. Pilot randomised controlled trial of a remotely delivered online intervention for adolescent mental health problems in India: lessons learned about low acceptability and feasibility during the COVID-19 pandemic. *BJPsych Open*. 2023;9(1):e7. doi:10.1192/bjo.2022.624

Gonsalves PP, Hodgson ES, Bhat B, et al. App-based guided problem-solving intervention for adolescent mental health: a pilot cohort study in Indian schools. *Evid Based Ment Health*. 2021b;24(1):11-18. doi:10.1136/ebmental-2020-300194

Huen JM, Lai ES, Shum AK, et al. Evaluation of a Digital Game-Based Learning Program for Enhancing Youth Mental Health: A Structural Equation Modeling of the Program Effectiveness. *JMIR Ment Health*. 2016;3(4):e46. doi:10.2196/mental.5656

López-Pérez B, Pacella D. Interpersonal emotion regulation in children: Age, gender, and cross-cultural differences using a serious game. *Emot Wash DC*. 2021;21(1):17-27. doi:10.1037/emo0000690

Ong JG, Lim-Ashworth NS, Ooi YP, et al. An Interactive Mobile App Game to Address Aggression (RegnaTales): Pilot Quantitative Study. *JMIR Serious Games*. 2019;7(2):e13242. doi:10.2196/13242

Pacella D, López-Pérez B. Assessing children’s interpersonal emotion regulation with virtual agents: The serious game Emodiscovery. *Comput Educ*. 2018;123:1-12. doi:10.1016/j.compedu.2018.04.005

Ros-Morente A, Cabello E, Filella G. Analysis of the Effects of two Gamified Emotional Education Software’s in Emotional and Well-being Variables in Spanish Children and Adolescents. *Int J Emerg Technol Learn IJET*. 2018;13:148. doi:10.3991/ijet.v13i09.7841

Sanchez R, Brown E, Kocher K, DeRosier M. Improving Children’s Mental Health with a Digital Social Skills Development Game: A Randomized Controlled Efficacy Trial of Adventures aboard the S.S. GRIN. *Games Health J*. 2017;6(1):19-27. doi:10.1089/g4h.2015.0108

Shum AK, Lai ES, Leung WG, et al. A Digital Game and School-Based Intervention for Students in Hong Kong: Quasi-Experimental Design. *J Med Internet Res*. 2019;21(4):e12003. doi:10.2196/12003

Tuijnman A, Kleinjan M, Olthof M, Hoogendoorn E, Granic I, Engels RC. A Game-Based School Program for Mental Health Literacy and Stigma on Depression (Moving Stories): Cluster Randomized Controlled Trial. *JMIR Ment Health*. 2022;9(8):e26615. doi:10.2196/26615
